# Coordination-based nanocomposite hydrogel promotes tissue regeneration under infection-compromised conditions

**DOI:** 10.1093/rb/rbag094

**Published:** 2026-05-12

**Authors:** Kailing Yu, Jia Zhong, Yilin Ma, Jia Li, Yinhui Wei, Hangsheng Zheng, Fanzhu Li, Lai Jiang

**Affiliations:** School of Pharmaceutical Sciences, Zhejiang Chinese Medical University, Hangzhou 31140, China; School of Pharmaceutical Sciences, Zhejiang Chinese Medical University, Hangzhou 31140, China; School of Pharmaceutical Sciences, Zhejiang Chinese Medical University, Hangzhou 31140, China; School of Pharmaceutical Sciences, Zhejiang Chinese Medical University, Hangzhou 31140, China; School of Pharmaceutical Sciences, Zhejiang Chinese Medical University, Hangzhou 31140, China; School of Pharmaceutical Sciences, Zhejiang Chinese Medical University, Hangzhou 31140, China; School of Pharmaceutical Sciences, Zhejiang Chinese Medical University, Hangzhou 31140, China; School of Pharmaceutical Sciences, Zhejiang Chinese Medical University, Hangzhou 31140, China

**Keywords:** daphnetin-copper nanocomposite gel, tissue regeneration, self-assembled nanoparticles, biofilm-associated infections, synergistic antibacterial mechanism, biofilm disruption

## Abstract

Impaired tissue regeneration, rather than mere bacterial colonization, represents the core pathological challenge in infected wounds, where persistent infection, biofilm formation and inflammatory dysregulation collectively disrupt the healing process. Herein, we report a coordination-based nanocomposite hydrogel (DAP-Cu NGs) designed to restore regenerative capacity by integrating infection control with active microenvironmental remodeling. The system comprises daphnetin-copper nanoparticles (DAP-Cu NPs) self-assembled via coordination chemistry and loaded into a poloxamer thermosensitive hydrogel. This design achieves two key objectives: nanoconfinement of Cu^2+^ to mitigate cytotoxicity, and pH-responsive release enabling spatiotemporally controlled drug delivery within the acidic infection microenvironment. Beyond synergistic elimination of pathogens and biofilms, DAP-Cu NGs actively modulate the regenerative niche by inducing macrophage polarization toward the pro-repair M2 phenotype, promoting keratinocyte and fibroblast migration, and enhancing angiogenesis with orderly collagen deposition. In an infected wound model, DAP-Cu NGs significantly accelerated wound closure and achieved near-complete tissue reconstruction with favorable biocompatibility. Critically, these regenerative outcomes were accomplished without exogenous growth factors, highlighting the inherent bioactivity of the coordination platform. This work establishes a paradigm shift from passive antimicrobial therapy toward active regeneration-engaging biomaterials, positioning infection control as an enabling step rather than a therapeutic endpoint for treating infection-compromised wounds.

## Graphical Abstract

**Graphical Abstract:**
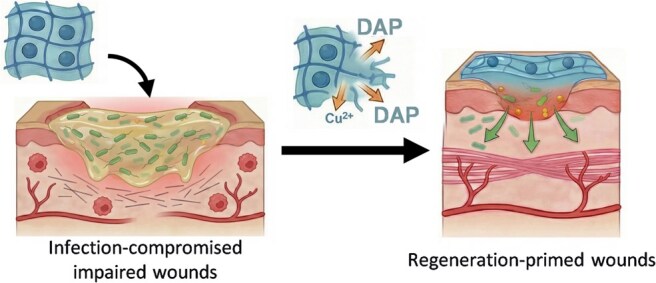

## Introduction

Impaired tissue regeneration represents the fundamental pathological challenge in infected wounds, where the healing process is disrupted not merely by the presence of pathogens, but by the complex interplay between persistent infection and dysregulated host repair mechanisms [1]. Unlike sterile wounds, infected wounds exhibit a sustained failure to transition through the normal healing phases, which is characterized by uncontrolled inflammation, inadequate angiogenesis and delayed matrix remodeling, ultimately leading to chronic non-healing and functional tissue loss. Traditional therapeutic approaches, primarily focused on pathogen elimination, often fail to restore the intrinsic regenerative capacity of the wound, highlighting the need for strategies that address both infection and the underlying regenerative deficit.

Bacterial colonization and biofilm formation are key instigators of this regenerative failure. By constructing physical barriers and modulating bacterial gene expression, biofilms enable pathogens to evade antibiotic clearance and host immune surveillance, perpetuating infection and treatment resistance [2–4]. Concurrently, the infected wound microenvironment exhibits pathological features including local acidosis, excessive accumulation of inflammatory mediators and immune dysregulation [5, 6]. These abnormalities not only support bacterial persistence but also directly impair essential regenerative processes such as cell migration, extracellular matrix remodeling and angiogenesis, thereby amplifying tissue damage and delaying repair.

To address these multifaceted challenges, various antibacterial strategies have been developed, including antibiotics, metal ions and natural bioactive compounds [7–10]. However, from a regenerative medicine perspective, existing approaches possess inherent limitations. Antibiotics often stay in the local area for a short time and are prone to rapid drug resistance. Although transition metal ions such as copper can play a broad-spectrum antibacterial role by destroying the bacterial membrane structure, interfering with protein function and inducing oxidative stress, the uncontrolled release of free copper ions can cause the risk of cytotoxicity and tissue damage [11–13]. Natural active molecules such as daphnetin (DAP), as coumarin derivatives, have antibacterial, anti-inflammatory and immunomodulatory functions, and can interfere with bacterial metabolism and signaling pathways, but their poor solubility and low bioavailability limit their application potential [14–17]. It is worth noting that copper ions also play an important regulatory role in angiogenesis and tissue remodeling, suggesting that if their controlled delivery can be achieved, they will be expected to be integrated into regenerative biomaterials. In summary, a single-component antibacterial system is difficult to meet the multiple requirements involved in tissue regeneration in an infected microenvironment.

In recent years, the progress of nanotechnology has promoted the rational design of biomaterials with both infection control and regeneration support functions. Among them, the coordination-driven self-assembly of metal-organic nanostructures provides an effective strategy for improving the stability and controlled release of antibacterial agents, and at the same time endows them with microenvironment-responsive drug release behavior, especially the pH-responsive characteristics that are highly matched with the acidic conditions of the infected wound [18–22]. At the same time, hydrogels have become a promising platform material in regenerative medicine due to their good biocompatibility, tunable physicochemical properties and the ability to form a hydrated three-dimensional network that supports cell infiltration and tissue remodeling. Therefore, integrating functional nanostructures into the hydrogel system provides a strong technical path for constructing regenerative biomaterials that can actively promote tissue repair under infected and damaged conditions.

In this study, we designed a coordination-based nanocomposite hydrogel (DAP-Cu NGs) with the explicit goal of restoring regenerative capacity in infected wounds by integrating infection control with active microenvironmental remodeling. The system comprises DAP-copper self-assembled nanoparticles (DAP-Cu NPs) loaded into a poloxamer thermosensitive hydrogel. DAP-Cu NPs were spontaneously formed through the coordination between DAP and Cu^2+^, which could realize the controlled release of copper ions and significantly enhance its antibacterial activity. The composite hydrogel can effectively inhibit bacterial growth and biofilm formation, thus creating a favorable microenvironment for tissue regeneration. The results showed that DAP-Cu NGs could synchronously promote key regenerative processes such as cell migration, collagen deposition and inflammation resolution, and accelerate wound closure in the infected wound model. At the same time, by controlling the release of copper ions, the system showed good *in vivo* biosafety.

In summary, this work develops a biomaterial strategy that can promote tissue regeneration under infection-damaged conditions by integrating coordination chemistry, nanostructure engineering and hydrogel delivery platform. The relevant findings provide an important reference for the rational design of multifunctional regenerative biomaterials with both anti-infection and pro-repair functions, and also provide new ideas for the clinical treatment of infectious and chronic wounds.

## Materials and methods

### Preparation of DAP-Cu NPs

To make a DAP stock solution of 100 mg/mL, 20 mg of DAP was weighed and dissolved in 200 μL of dimethyl sulfoxide (DMSO). To make a 10 mg/mL CuCl_2_ stock solution, 10 mg of copper chloride dihydrate (CuCl_2_·2H_2_O) was weighed and dissolved in 2 mL of ultrapure water. A total of 50 μL of DAP stock solution was diluted within 0.95 mL of ultrapure water. After stirring evenly, 1 mL of 10 mg/mL CuCl_2_ stock solution (the molar ratio of DAP to CuCl_2_ was 1:2) was added. Then, NaOH solution with a pH of 12 was added dropwise to adjust the pH to 8 and stirred overnight at 25°C. The next day, the DAP-Cu nanoparticles were collected by centrifugation at 12 000 rpm for 10 min, washed alternately with anhydrous ethanol and ultrapure water for three times, and finally dispersed with ultrapure water. All preparations were performed in triplicate independent experiments.

### Characterization of DAP-Cu NPs

DAP-Cu NPs were diluted 20 times with ultrapure water and dropped onto a 200-mesh copper grid. After drying, the morphology was observed by transmission electron microscope (TEM, JEOL JEM-F200). An appropriate amount of DAP-Cu NPs was dispersed in 1 mL of ultrapure water, and the hydrated particle size, polydispersity index (PDI) and zeta potential were measured by a Malvern laser particle size analyzer (Nano-ZS90). DAP solution, CuCl_2_ solution, a physical mixture of DAP and CuCl_2_ (DAP&CuCl_2_), and DAP-Cu NPs solution at 10 μg/mL were each placed into a quartz cuvette, and their ultraviolet visible absorption spectra were measured in the wavelength range of 300−800 nm. Appropriate amounts of DAP, CuCl_2_, DAP&CuCl_2_ and DAP-Cu NPs were weighed, and their FTIR spectra were scanned and recorded by Fourier transform infrared spectrometer (Nicolet Is50). The semi-quantitative analysis of C, H and Cu elements in DAP-Cu NPs was carried out by energy dispersive X-ray spectroscopy (EDS). The atomic composition and valence state of DAP-Cu NPs were analysed by X-ray photoelectron spectroscopy (XPS, K-Alpha). In the stability study, an appropriate amount of the sample was dispersed in ultrapure water and stored at 4°C. The particle size, PDI and zeta potential were measured on days 0, 2, 4, 6, 8, 10 and 15. For each characterization, measurements were performed on three independently prepared batches of nanoparticles.

### The encapsulation efficiency and drug loading of DAP-Cu NPs

The HPLC methodology of analysis for DAP was developed as follows: an Athene C18-BIO column (150 mm × 4.6 mm, 5 μm, 300 Å) was used, the mobile phase was acetonitrile–0.5% glacial acetic acid (25:75), the injection volume was 10 μL, the column temperature was 25°C, the flow rate was 1 mL/min and the detection wavelength was 327 nm. For the preparation of the standard solution, 3.00 mg of DAP standard was accurately weighed, placed in a 5 mL volumetric flask, 2 mL of anhydrous ethanol was added and then dissolved by ultrasonication. Then, anhydrous ethanol was added to obtain a 600 μg/mL DAP standard stock solution. The DAP standard stock solution was aspirated and diluted with anhydrous ethanol to prepare a series of solutions of 120, 60, 30, 20, 6 and 1.2 μg/mL. After filtration through a 0.22 μm microporous membrane, the filtrate was sampled under the above chromatographic conditions, the peak area was recorded and the peak area was used as the ordinate and the concentration as the abscissa for linear regression.

Lyophilized DAP-Cu NPs powders from three different batches were each dispersed in 3 mL of anhydrous ethanol by sonication. The dispersions were then transferred to 5 mL volumetric flasks and diluted to volume. After low‑temperature centrifugation at 12 000 rpm for 15 min, the supernatants were withdrawn and filtered through 0.22 µm microporous membranes. The filtrates were injected into the chromatographic system under the specified conditions, and the concentration of DAP was calculated using a standard curve. Finally, the encapsulation efficiency (EE%) and drug loading (DL%) were calculated according to the following formula. Three independent batches of DAP-Cu NPs were analysed


$$\text{DL}(\%)=\frac{\mathrm{The}\;\mathrm{loaded}\;\mathrm{drug}\;\mathrm{mass}}{\mathrm{The}\;\mathrm{total}\;\mathrm{mass}\;\mathrm{of}\;\mathrm{nanoparticles}}\times 100\%$$

$$\text{EE}(\%)=\frac{\mathrm{The}\;\mathrm{loaded}\;\mathrm{drug}\;\mathrm{mass}}{\;\mathrm{The}\;\mathrm{total}\;\mathrm{drug}\;\mathrm{mass}}\times 100\%$$

### Molecular dynamics simulation of DAP-Cu NPs

The 3D structure of DAP was generated by OpenBabel (version 3.1.1), and optimized by the steepest descent method, 1500 iterations and MMFF94 force field. First, 30 DAP molecules and 15 Cu^2+^ ions were evenly placed in the simulation box by Packmol. Then, the simulation system was constructed using Gromacs 2021.7; the GAFF force field was adopted, the water model was TIP3P; Na^+^ and Cl^−^ ions were used to balance the system charge; the steepest descent method was used to minimize the energy of the system, and the NVT and NPT equilibrium of the system was carried out for a short time under constant temperature (25°C) and constant pressure (1 Bar). After the temperature and density of the system were stable, the molecular dynamics simulation (100 ns) was carried out, and the trajectory data were collected. Finally, the motion trajectory was analysed, the Leapfrog algorithm was used to integrate the Newton motion equation, the integration time step was 2 fs, and the root mean square deviation (RMSD), the radius of gyration (Rg), the hydrogen bond and the interaction energy were plotted as a function of time.

### *In vitro* release behavior of DAP-Cu NPs

To investigate the *in vitro* release behavior of DAP-Cu NPs, release media with pH values of 7.4, 6.5 and 5.3 were selected to simulate the normal environment and the microenvironment of infected wounds, respectively. The constant temperature air bath oscillator was preset to 100 rpm and 32 ± 0.5°C. 2 mL each of DAP solution and DAP-Cu NPs solution were accurately pipetted. Three parallel samples were prepared for each solution, and each sample was transferred into a dialysis bag (MWCO 3500 Da). The dialysis bags were immersed in 50 mL centrifuge tubes containing 30 mL of the corresponding pH medium preheated to 32°C, and the centrifuge tubes were immediately placed in the shaker. Samples of 1 mL were taken at 5, 15, 30, 45 min and 1, 2, 4, 6, 8, 12, 24 h, respectively, and the same amount of medium at 32°C was quickly added. The samples at each time point were filtered through a 0.22 μm microporous membrane, and the filtrate was taken and injected for detection according to the chromatographic conditions set in the previous text. The *in vitro* cumulative release rate of DAP was calculated according to the following formula


$$Q_{t}(\%)=\frac{C_{t}\times V+\sum_{i=1}^{t-1}C_{i\times }V_{i}}{W_{0}}\times 100\%$$

where *C_t_* is the drug concentration measured at the *t* time point, *V* is the total volume of the release medium (30 mL), *V_i_* is the volume of the sample taken at the *i*th time point (1 mL) and *W*_0_ is the total drug loading. All release experiments were performed in triplicate for each pH condition.

### Preparation, characterization and *in vitro* drug release behavior of DAP-Cu NGs

To prepare the blank hydrogel, 20 mL of poloxamer 407 solution with a mass–volume ratio (W/V) of 18.0%, 18.5%, 19.0%, 19.5% and 20% were prepared, respectively, and the phase transition temperature was measured. To prepare the nanoparticles-loaded thermosensitive hydrogel (DAP-Cu NGs), 1.85 g of poloxamer 407 was weighed, 9 mL of ultrapure water was added and the mixture was placed at 4°C overnight and stirred until completely dissolved. In addition, 4 mg of DAP-Cu NPs was weighed and dispersed in 1 mL of ultrapure water, stirred evenly and mixed with the above poloxamer 407 solutions to obtain a DAP-copper nanocomposite gel (DAP-Cu NGs). The preparation of DAP Gel and CuCl_2_ Gel was the same as that of DAP-Cu NGs. The temperature of the drug-loaded gel was scanned in oscillation mode. The temperature was set to start from 25°C and increased to 40°C at a rate of 0.5°C/min. The relationship between *η* (viscosity), *G*′ (storage modulus) and *G*″ (loss modulus) of each group of gels with temperature was measured. The DAP-Cu NGs were freeze-dried, cut with a blade, placed on a conductive adhesive and sprayed with gold. Then, they were observed and recorded by scanning electron microscope (SEM, SU8010). The *in vitro* release behavior of DAP-Cu NGs was investigated by dialysis bag method. The DAP Gel group and DAP-Cu NGs group were set up in the experiment, and the specific operation was the same as described above.

### Bacteria strains and cell lines

*Escherichia coli* (*E. coli* DH-5α) and *Staphylococcus aureus* (*S. aureus* ATCC 43300) were selected as representative Gram-negative and Gram-positive bacteria, respectively, as both species are among the most frequently isolated pathogens in clinical wound infections, including surgical site infections and chronic wounds. Although *E. coli* DH-5α is a laboratory strain, it is widely used in antibacterial studies due to its genetic stability and reproducibility, providing a reliable model for evaluating antibacterial performance.

Human immortalized keratinocytes (HaCaT), mouse fibroblasts (L929) and mouse macrophages (RAW264.7) were employed as well-established *in vitro* models to represent key cellular components involved in cutaneous wound healing. Specifically, HaCaT cells were used to evaluate re-epithelialization processes (keratinocyte proliferation and migration), L929 cells to assess extracellular matrix remodeling (fibroblast function) and RAW264.7 cells to investigate inflammatory regulation (macrophage activation and polarization). These cell lines are widely used in skin regeneration and wound healing studies to model the major biological processes involved in tissue repair. All cell lines were purchased from Beijing Beina Chuanglian Biotechnology Research Institute.

### Determination of minimum inhibitory concentration and minimum bactericidal concentration

The minimum inhibitory concentration (MIC) and minimum bactericidal concentration (MBC) of DAP and CuCl_2_ against *S. aureus* and *E. coli* were determined by microbroth dilution method. A single colony was picked from the bacterial culture dish and placed in a tube containing fresh Tryptic Soy Broth (TSB) and cultured in a constant temperature shaker at 37°C and 200 r/min for 18 h. The next day, an appropriate amount of bacterial solution was transferred to fresh TSB for activation for 2 h, and diluted to 1 × 10^6^ CFU/mL for later use. 100 μL of the sample stock solution was added to a 96-well plate. A series of gradient concentration drug solutions was prepared by 2-fold dilution. An appropriate amount of diluted bacterial solution was added to each well (three parallel samples were set for each group and control wells are set). After sealing, the plate was placed at 37°C for 18 h. After incubation, the OD_600_ value was measured, and the lowest drug concentration equivalent to the absorbance value of the negative control was taken as MIC; the bacterial solution of MIC and the first two gradient concentrations were taken for colony counting, and the lowest concentration without bacterial growth was taken as MBC.

### Synergistic antibacterial experiment

The synergistic antibacterial experiment was carried out by checkerboard method to determine the synergistic antibacterial concentration of DAP and copper chloride against *S. aureus* and *E. coli*. The activated bacterial suspension was prepared according to the method described above. The DAP solution (A) and CuCl_2_ solution (B) with 4096 µg/mL were Prepared. The DAP solution was diluted horizontally in the 96-well plate, while the CuCl_2_ solution was diluted vertically. Then, 100 μL of bacterial solution was added and the plate was placed at 37°C for 18 h. After incubation, the MIC_AB_ of the combined system was determined by bacterial counting. The fractional inhibitory concentration (FIC) index was calculated according to the following formula:


$$\text{FIC}=\frac{{\text{MIC}}_{\mathrm{AB}}}{{\text{MIC}}_{A}}+\frac{{\text{MIC}}_{\mathrm{AB}}}{{\text{MIC}}_{B}}$$

### Antibacterial experiment of DAP-Cu NPs

The antibacterial activity of DAP-Cu NPs against *S. aureus* and *E. coli* was determined by microbroth dilution method. The experimental method was the same as above. The concentration of DAP-Cu NPs was calculated according to the content of DAP in the nanoparticles. Experiments were repeated three times independently.

### Antibacterial experiment of DAP-Cu NGs

The antibacterial properties of DAP-Cu NGs were tested by the zone of inhibition method. The bacteria were activated as described above, and the bacterial solution was adjusted to 1 × 10^8^ CFU/mL. The bacterial solution was evenly spread on the surface of TSA. After it was slightly dried, the TSA was punched with an Oxford cup. The Blank Gel, DAP Gel, CuCl_2_ Gels, DAP&CuCl_2_ Gel and DAP-Cu NGs were added to the holes. The plates were placed in an incubator and cultured at 37°C for 24 h. The size of the inhibition zone formed around different hydrogel sheets was observed, and the diameter of the inhibition zone was recorded.

In order to further explore the antibacterial activity of hydrogels, different types of hydrogels were co-incubated with *E. coli* and *S. aureus*, respectively. About 100 µL of Blank Gel, DAP Gel, CuCl_2_ Gel, DAP&CuCl_2_ Gel and DAP-Cu NGs were added to the sterile 96-well plate. After the hydrogel was completely solidified in the 37°C incubator, 100 µL of bacterial solution was added to each well. The plate was placed in a 37°C incubator for 24 h, and the contents of each well were taken out the next day, and the bacteria were counted after gradient dilution with PBS.

The morphological changes of bacteria were observed by SEM. The treated bacterial solution was collected, centrifuged at 3000 rpm for 10 min and the supernatant was discarded. Glutaraldehyde was added for fixation for 30 min, and then washed with ultrapure water three times, each time for 10 min. After washing, the samples were dehydrated with 50%, 60%, 70%, 80%, 90% and 100% ethanol in turn, and then dehydrated with 50% and 100% tert-butanol for 10 min each. The treated samples were dried in a vacuum freeze dryer, and SEM images were taken after gold spraying.

### *In vitro* antibacterial biofilm experiment of DAP-Cu NGs

The *in vitro* antibacterial biofilm effect of DAP-Cu NGs was studied by crystal violet staining method. Activated bacteria was inoculated into a sterile 24-well plate, and fresh TSB was replaced every 12 h, and the mature biofilm was constructed by continuous culture for 48 h. Then, the biofilms were treated with PBS, Blank Gel, DAP Gel, CuCl_2_ Gel, DAP&CuCl_2_ Gel and DAP-Cu NGs, respectively, and placed in a 37°C incubator for 24 h. After incubation, the biofilms were washed three times with PBS, and then fixed with 4% paraformaldehyde for 30 min. About 0.1% (w/v) crystal violet dye was then added to each well, and the cells were stained at room temperature for 20 min, and washed with ultrapure water for three times. After drying again, 33% glacial acetic acid was added to each well to completely dissolve the crystal violet, and the absorbance at a wavelength of 570 nm was measured, and the biofilm inhibition rate was calculated accordingly. In addition, the same experimental steps were used to stain the biofilm with BacLight^®^ dye (B34950, Invitrogen™) after the culture, and the biofilm morphology was observed and photographed with a confocal laser scanning microscope (CLSM, Zeiss LSM880). The assay was performed with three independent biological replicates (*n* = 3).

### Cytotoxicity assay

The MTT method was used to evaluate the toxicity of free DAP, CuCl_2_ and DAP-Cu NPs to L929 cells, HaCaT cells and RAW264.7 cells. Cells in the logarithmic growth phase were taken, digested with trypsin and counted. The cells were inoculated into 96-well plates at a density of 1 × 10^4^ cells per well. Blank wells were set as controls and the plates were placed in a constant temperature incubator at 37°C and 5% CO_2_ for overnight culture. The culture medium was discarded, and the sample groups were added with free DAP, CuCl_2_ and DAP-Cu NPs at gradient concentrations, respectively. The negative control group was only added with complete culture medium, and co-incubated with cells for 24 h. The medium was discarded, and 100 µL of MTT working solution was added to each well under dark conditions, and incubated in a constant temperature incubator for 4 h. After incubation, the medium was removed, and 150 µL of DMSO was added to each well. The absorbance of each well was measured at 490 nm using a microplate reader (SpectraMax M2e), the cell survival rate and half inhibitory concentration (IC_50_) were calculated. The assay was performed with three independent biological replicates (*n* = 3).

### Cell migration assay

The effect of DAP-Cu NPs on cell migration was investigated using L929 and HaCaT cells. The two types of cells in the logarithmic growth phase were taken, digested with trypsin and counted. Then, 5 × 10^5^ cells were inoculated into each well of a 6-well plate and placed in a constant temperature incubator at 37°C and 5% CO_2_ for culture. The cells were scraped off by drawing a line in each well with a 200 μL pipette tip perpendicular to the pre-drawn horizontal line. The sample group was added with free DAP, CuCl_2_, DAP&CuCl_2_ and DAP-Cu NPs, respectively, and the negative control group was added with blank culture medium. The cell migration of each group was observed and photographed with a Nikon inverted microscope (Nikon Ti-s) immediately after administration and after 24 h of culture. The images were processed with ImageJ and the area was measured to calculate the cell migration rate. Experiments were repeated three times independently.

### Inflammation regulation ability assessment

RAW264.7 cells were used to investigate the anti-inflammatory regulatory effect of DAP-Cu NPs. RAW264.7 cells in the logarithmic growth phase were collected and then inoculated into 6-well plates at 5 × 10^5^ cells per well. Except for the blank group, complete medium containing 1 µg/mL LPS was added to the remaining wells and incubated overnight in a constant temperature incubator at 37°C and 5% CO_2_. The next day, the original culture medium was discarded, and the sample groups were added with culture medium containing free DAP, CuCl_2_, DAP&CuCl_2_ and DAP-Cu NPs, respectively. The blank control group and the positive control group were added with conventional cell culture medium, and incubated for 24 h. After incubation, the culture medium of each group was collected, centrifuged at 3000 rpm for 20 min, and the supernatant was taken and stored at −20°C for later use.

The levels of TNF-α, iNOS, IL-6 and IL-1β in the supernatant were detected by ELISA. Gradient concentrations of standards were added to the standard wells, no liquid was added to the blank wells and cell supernatants of each group were added to the sample wells. Except for the blank wells, the remaining wells were added with horseradish peroxidase-labeled detection antibody and incubated in a 37°C incubator for 60 min in the dark. After incubation, the wells were washed five times, substrate mixture was added to all wells, incubated in the dark in a 37°C incubator for 15 min, Then stop solution was added, the OD value was measured at 450 nm wavelength with an microplate reader and the final concentration of each group of samples was calculated according to the standard curve equation. All ELISA measurements were performed in triplicate.

### Bioinformatics analysis

The transcriptomics data generated by the Illumina platform are used for bioinformatics analysis. All analyses were performed on the Majorbio Cloud Platform (www.majorbio.com).

### Animal care

Female Balb/c mice aged 6–8 weeks old were ordered from the Spfbiotech (Suzhou) Co., Ltd. The animal experiments complied with the guidelines of the Cocalero Biotech (Hangzhou) Co., Ltd. Ethics Committee (KKN-2025-071601). All *in vivo* experiments were conducted with appropriate blinding procedures. Outcome assessments, including wound measurement, histological evaluation and hematological analysis, were performed by investigators blinded to group allocation.

### Establishment of a full-thickness skin infectious wound model in mice

Female Balb/c mice (female, 6–8 weeks old, 16–24 g) were used in the experiment. Randomization was performed prior to treatment allocation using a random number generator. About 0.3% pentobarbital sodium was prepared and mice were anesthetized by intraperitoneal injection (0.2 mL/10 g). The hair on the back area was shaved, and a circular skin with a diameter of 10 mm was removed from the skin on the back with a biopsy punch. About 50 µL of *E. coli* suspension (10^6^ CFU/mL) was evenly dropped on the wound site to create a full-thickness skin wound infection model. The mice were randomly assigned to six groups: model group (equal volume saline), Blank Gel, DAP Gel, CuCl_2_ Gel, DAP&CuCl_2_ Gel and DAP-Cu NGs. After modeling, the mice were administered once every other day for 15 days. During the treatment, the mice were free to eat and drink.

### Healing of infected wounds and bacterial colonies in wounds of mice

During the drug treatment, the wound sites of mice were photographed and observed on days 0, 3, 6, 9, 12 and 15. Wound area measurements were performed by two independent investigators blinded to group allocation using ImageJ software, with the scale calibrated according to the actual length of the caliper to calculate the wound area and healing rate. On the third day of drug treatment, the exudate from the wound site was taken, and the colony number was calculated by the plate counting method. After the treatment, the skin of the wound site of each group of mice was taken, fixed by soaking in 4% paraformaldehyde, embedded in paraffin, sliced and stained with Masson, H&E and IHC to evaluate the pathological changes of the pathological site. Immunohistochemical analyses were evaluated by two independent investigators blinded to the treatment groups.

### Biosafety assessment

The whole blood of mice was collected and placed in a sodium heparin anticoagulant tube. The red blood cells (RBCs) were precipitated by centrifugation at 1500 rpm for 15 min. The RBCs were washed with saline until the supernatant was colorless. About 200 µL of RBC precipitate was taken and mixed with 9.8 mL of saline to obtain a 2% (v/v) RBC suspension. The RBC suspension was mixed with saline (negative control group), pure water (positive control group), CuCl_2_, DAP, DAP&CuCl_2_, DAP-Cu NPs, Blank Gel, CuCl_2_ Gel, DAP Gel, DAP&CuCl_2_ Gel and DAP-Cu NGs. The mixed solution was incubated in a 37°C incubator for 1 h, and the samples were centrifuged. The absorbance of the supernatant at 540 nm was measured with an microplate reader, and the hemolysis rate was calculated.

During the drug treatment, the weight of each group of mice was recorded every 2 days from the day of modeling, and the weight change curve of each group of mice was drawn for 15 days to evaluate the weight change of each group of mice during the treatment.

After the end of the treatment cycle, the whole blood of each group of mice was collected into 1.5 mL EDTA-K2 anticoagulant tubes. About 200 μL of blood was taken and measured by automated hematology analyzer. After the end of the treatment cycle, the mice were killed by CO_2_, and the heart, liver, spleen, lung and kidney of the mice were taken out and fixed in 4% paraformaldehyde fixative. After dehydration in gradient ethanol, the paraffin was embedded and sliced. The histopathological changes of each organ were observed by H&E staining. All assays were performed with three independent biological replicates. Hematological analysis was performed by personnel blinded to group allocation. Histopathological evaluation of organ sections was conducted by a certified pathologist blinded to treatment conditions.

### Statistical analysis

Sample sizes were determined based on standard practices in the field and preliminary experiments, and were sufficient to detect statistically significant differences between the groups. The data were presented as means ± SD (standard deviations). Two-tailed Student’s *t*-test was used for two groups comparison, and multiple group comparison was analysed by one-way analysis of variance (ANOVA). *P < 0.05 was considered as significant statistical difference. The statistical analysis was performed using GraphPad Prism 10.4.2 software.

## Results

### The self-assembly property of DAP-Cu NPs endows controlled release and stability

**Figure 1:**
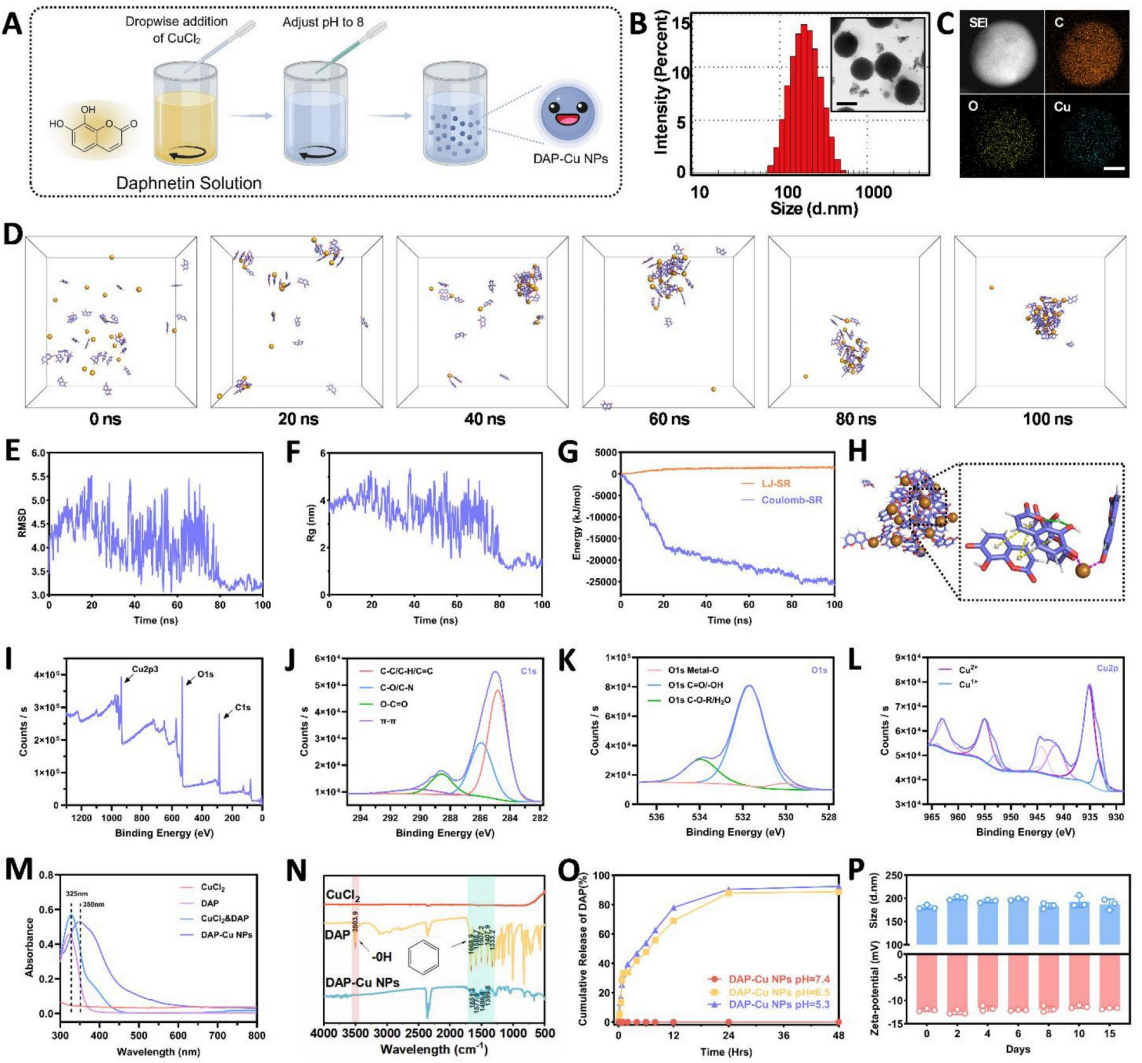
(**A**) Schematic diagram of the preparation of DAP-Cu NPs. Created in BioRender. *Jiang, L.* (2026) (**B**) Particle size of DAP-Cu NPs with the inserted TEM image. Scale bar = 200 nm. (**C**) Elemental distribution in DAP-Cu NPs. Scale bar = 50 nm. (**D**) Trajectory screenshots of molecular simulation that was used to show the self-assembly process of DAP and copper ions. (**E**) RMSD variation curve of DAP and copper ion during self-assembly. (**F**) Rg variation curve of DAP and copper ion during self-assembly. (**G**) The change curves of the van der Waals interaction energy (LJ-SR) and the Coulomb interaction energy (Coulomb-SR) between molecules in the short-range during the self-assembly process of DAP and copper ions. (**H**) Analysis of the interaction between DAP and copper ions. (**I**) XPS patterns of DAP-Cu NPs and high-resolution of C 1s (**J**), O 1s (**K**) and Cu 2p (**L**) peaks. (**M**) UV–Vis spectra of DAP, CuCl_2_, DAP&CuCl_2_ and DAP-Cu NPs. (**N**) FTIR spectra of DAP, CuCl_2_ and DAP-Cu NPs. (**O**) Release curves of DAP-Cu NPs in different media. (**P**) Stability test of DAP-Cu NPs.

We successfully prepared DAP-Cu NPs by a mild method (Figure 1A) and systematically analysed them by various characterization methods. The DLS results showed that the particle size distribution of DAP-Cu NPs was mainly concentrated around 100 nm (Figure 1B), with good dispersibility and appropriate size. TEM further confirmed its spherical structure, and the uniform existence of copper element was verified by the element distribution map, indicating that copper ions successfully participated in the assembly process (Figure 1C).

To further explore the stability and mechanism of self-assembly, molecular dynamics simulations were performed to investigate the self-assembly process (Figure 1D–H). As shown in Figure 1E and F, the RMSD and Rg values fluctuated during the initial 80 ns of simulation and subsequently stabilized, indicating structural equilibration of the assembled system. Analysis of interaction energies revealed an average LJ-SR energy of +1151.98 kJ/mol and an average Coulomb-SR energy of −18661.61 kJ/mol during the simulation (Figure 1G). The final simulation snapshot revealed π–π stacking interactions between DAP molecules, hydrogen bonding and coordination bonds between DAP and Cu^2+^ (Figure 1H).

XPS results further revealed the chemical bonding mechanism. It showed Cu 2p spectra with predominant Cu^2+^ signals and a minor Cu^1+^ component (Figure 1I and L). The C 1s spectrum displayed peaks corresponding to C–O and C=O groups (Figure 1J), and the O 1s spectrum exhibited a metal–oxygen peak at 532.6 eV, confirming Cu–O bond formation (Figure 1K). Ultraviolet–Visible (UV–Vis) spectroscopy revealed a red shift in the absorption peak of DAP upon coordination with Cu^2+^ (Figure 1M). FTIR spectra showed shifts in characteristic peaks of DAP after nanoparticle formation (Figure 1N). *In vitro* release studies revealed that DAP-Cu NPs exhibited a pH-responsive release behavior, with minimal release under neutral conditions (pH 7.4), while a rapid and pronounced release was observed under acidic conditions (pH 5.3 and 6.5) (Figure 1O). This accelerated release under acidic environments suggests a protonation-induced disruption of coordination interactions, leading to rapid nanoparticle dissociation (Figure 1O). Stability tests over 15 days showed no significant changes in particle size or PDI (Figure 1P).

### Synergistic effect of DAP&Cu^2+^ drives the enhanced antibacterial mechanism

**Figure 2:**
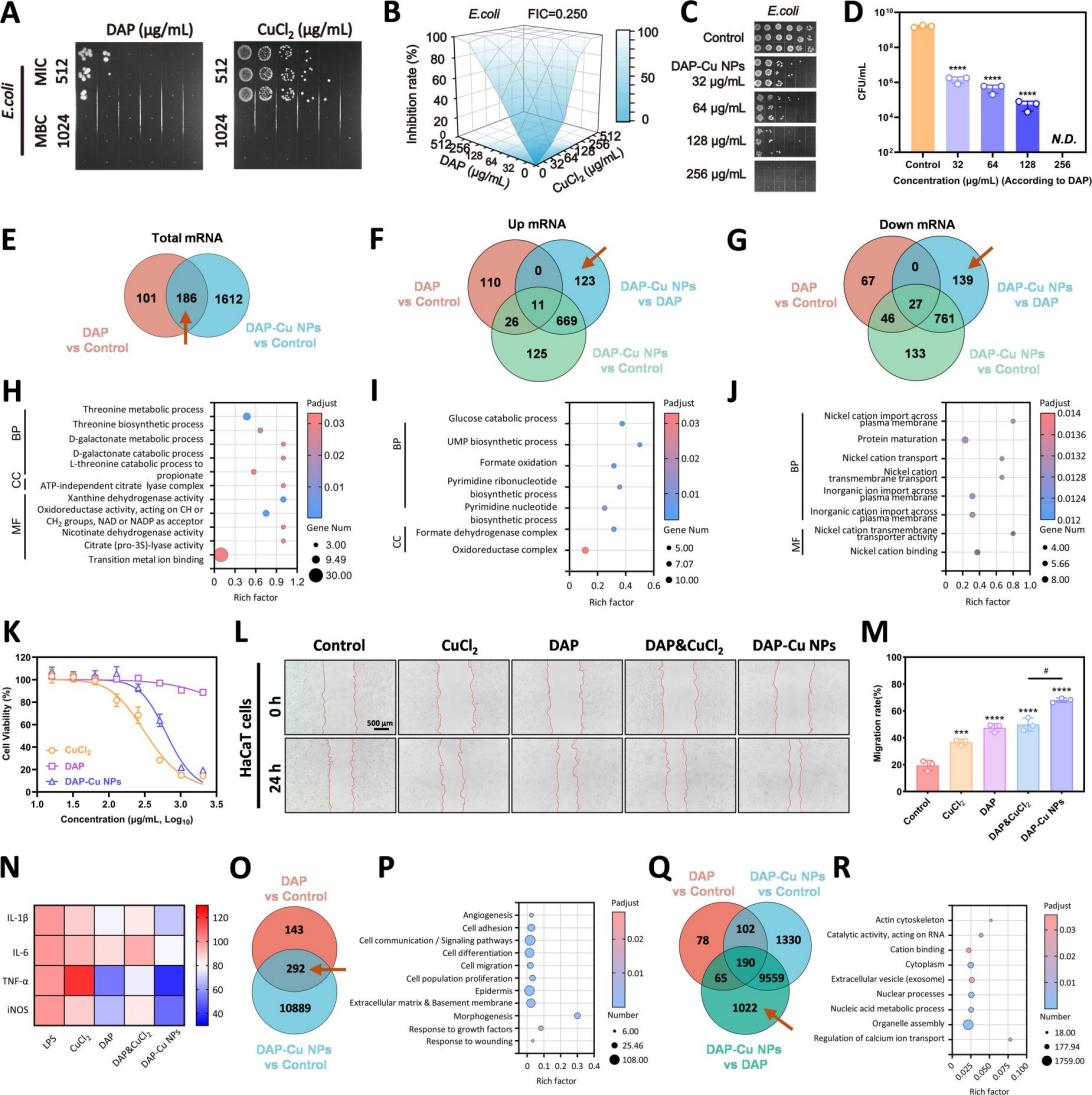
(**A**) The antibacterial effects of DAP and Cu^2+^ on *E. coli*. (**B**) Synergistic antibacterial results of DAP and Cu^2+^ on *E. coli*. (**C and D**) The antibacterial results of DAP-Cu NPs on *E. coli*, shown in the photos (**C**) and quantitative analysis (**D**). (**E–J**) Transcriptome analysis of the effects of DAP-Cu NPs on *E. coli*. (**E and H**) Venn analysis of DEGs in the transcriptome affected by DAP and DAP-Cu NPs, and GO enrichment analysis of the intersection. (**F and I**) Venn analysis of up-regulated DEGs in the transcriptome affected by DAP and DAP-Cu NPs, and GO enrichment analysis of the DAP-Cu NPs specific part. (**G and J**) Venn analysis of down-regulated DEGs in the transcriptome affected by DAP and DAP-Cu NPs, and GO enrichment analysis of the DAP-Cu NPs specific part. (**K**) IC_50_ curves of DAP, CuCl_2_ and DAP-Cu NPs for HaCaT cells. (**L**) Photographs of the migration experiment of HaCaT cells with DAP, CuCl_2_, DAP&CuCl_2_ and DAP-Cu NPs, and (**M**) quantitative analysis of migration rate. (**N**) The regulation results of DAP, CuCl_2_, DAP&CuCl_2_ and DAP-Cu NPs on inflammatory factors of LPS-induced RAW264.7 cells. (**O–R**) Transcriptome regulation analysis of DAP-Cu NPs on HaCaT cells. (**O and P**) Venn analysis of DEGs in the transcriptome affected by DAP and DAP-Cu NPs, and GO enrichment analysis of the intersection. (**Q and R**) Venn analysis of all DEGs in the transcriptome affected by DAP and DAP-Cu NPs, and GO enrichment analysis of the DAP-Cu NPs specific part. All data are expressed as mean ± SD (*n* = 3 per group). Statistical analysis was performed by two-tailed Student’s *t*-test (#*P* ≤ 0.05) and one-way ANOVA (*P ≤ 0.05, ***P ≤ 0.001 and ****P ≤ 0.0001).

First, we determined the baseline antibacterial activity of the single components against *E. coli*. It showed that the MBCs of DAP and CuCl_2_ against *E. coli* were both 1024 μg/mL (Figure 2A), indicating that both DAP and Cu^2+^ had weak antibacterial activity at low concentrations. Checkerboard assay revealed a FIC index of 0.25 for the combination of DAP and Cu^2+^ against *E. coli* (Figure 2B). Similar synergistic effects were observed against *S. aureus* (Supplementary Figure S1A and B). We further evaluated the antibacterial efficacy of DAP-Cu NPs. Co-incubation experiments showed that the colony forming units (CFUs) decreased in a dose-dependent manner, while the MBC (256 μg/mL) of the DAP-Cu NPs was only 1/4 of that of DAP alone (Figure 2C and D). This result indicates that DAP-Cu NPs significantly enhanced their antibacterial efficacy under synergistic action.

To elucidate the molecular mechanism, prokaryotic transcriptome analysis was performed on *E. coli* treated with DAP or DAP-Cu NPs (Supplementary Figure S2A–C). Venn diagram analysis identified 186 overlapping differentially expressed genes (DEGs) between the DAP and DAP-Cu NPs treatment groups (Figure 2E, the area indicated by the arrow), suggesting that the nano-modification did not change the core antibacterial mechanism of DAP. GO enrichment analysis showed that these genes were mainly concentrated in threonine metabolism, D-galactose metabolism, citric acid lysis and metal ion binding pathways, involving amino acid synthesis, sugar metabolism and metal enzyme activity regulation (Figure 2H). KEGG enrichment results further showed that it mainly acted on metabolic networks such as galactose metabolism, cysteine and methionine metabolism, and branched-chain amino acid synthesis (Supplementary Figure S2D).

Compared with DAP alone, DAP-Cu NPs induced 123 unique up-regulated genes (Figure 2F, pointed by arrow). GO enrichment analysis showed that these genes were mainly involved in glucose catabolism, formate oxidation and nucleotide biosynthesis (Figure 2I). KEGG enrichment showed that it was significantly involved in pyrimidine metabolism, arachidonic acid metabolism and tryptophan metabolism (Supplementary Figure S2E). On the other hand, DAP-Cu NPs specifically down-regulated 139 genes (Figure 2G, indicated by arrow). GO enrichment showed that these genes were related to transmembrane transport of nickel ions, inorganic cation transport and metal ion binding (Figure 2J). KEGG enrichment results revealed that it acted on key pathways such as pentose and glucuronic acid metabolism, ascorbic acid metabolism, fructose and mannose metabolism, and DNA nucleotide excision repair (Supplementary Figure S2F).

### DAP-Cu NPs simultaneously achieve wound microenvironment remodeling

Cytotoxicity of DAP, CuCl_2_ and DAP-Cu NPs was evaluated in HaCaT, L929 and RAW264.7 cells. DAP exhibited low toxicity within the effective antibacterial concentration range, while CuCl_2_ showed pronounced cytotoxicity with IC_50_ values of 343.6 μg/mL (HaCaT), 250.6 μg/mL (L929) and 114.6 μg/mL (RAW264.7). All substantially lower than its MBC (1024 μg/mL) (Figure 2K, Supplementary Figure S3A and B). In contrast, DAP-Cu NPs displayed a right-shifted dose–response curve with significantly increased IC_50_ values across all three cell lines (Supplementary Figure S3C).

Cell migration was assessed using HaCaT and L929 scratch assays. The scratch width in the control group remained nearly unchanged over 24 h. CuCl_2_ or DAP treatment alone resulted in limited migration, while the DAP& CuCl_2_ combination showed slight improvement. Notably, DAP-Cu NPs treatment led to the most pronounced scratch closure (Figure 2L, Supplementary Figure S3D), with migration rates significantly higher than those of other treatment groups (Figure 2M, Supplementary Figure S3E). In terms of inflammatory regulation, we detected the secretion level of inflammatory factors in RAW264.7 cells (Figure 2N, Supplementary Figure S3F). The results showed CuCl_2_ treatment failed to suppress the inflammatory response, whereas DAP significantly down-regulated the levels of IL-1β, TNF-α.

Combined with the results of the previous cell migration experiment, it can be inferred that DAP-Cu NPs have the dual function of promoting repair and alleviating the inflammatory microenvironment, which helps the wound to smoothly transition from the inflammatory phase to the proliferation phase. This advantage has potential value for the treatment of chronic or refractory wounds.

To further elucidate its molecular mechanism, transcriptome analysis was performed on HaCaT cells. We first compared the DEGs of the DAP and DAP-Cu NPs treatment groups (Supplementary Figure S3G–I). The results showed that there were a considerable number of DEGs between the two groups (Figure 2O), which were involved in the key links of cell repair and tissue regeneration. GO enrichment analysis showed that these co-regulated genes were significantly enriched in biological processes highly related to wound healing (Figure 2P, Supplementary Table S1), including cell adhesion and migration, cell differentiation, extracellular matrix and basement membrane remodeling, and angiogenesis. On this basis, DAP-Cu NPs induced a larger set of unique DEGs with broader pathway enrichment (Figure 2Q). GO enrichment results showed that these genes were mainly concentrated in cytoskeleton remodeling, nucleic acid metabolism and transcriptional regulation, organelle assembly, transmembrane transport of inorganic ions and calcium ion homeostasis maintenance (Figure 2R, Supplementary Table S2). These pathways were not significantly enriched in the DAP monotherapy group.

### DAP-Cu NGs significantly inhibited biofilm formation and destroyed the bacterial structure

**Figure 3:**
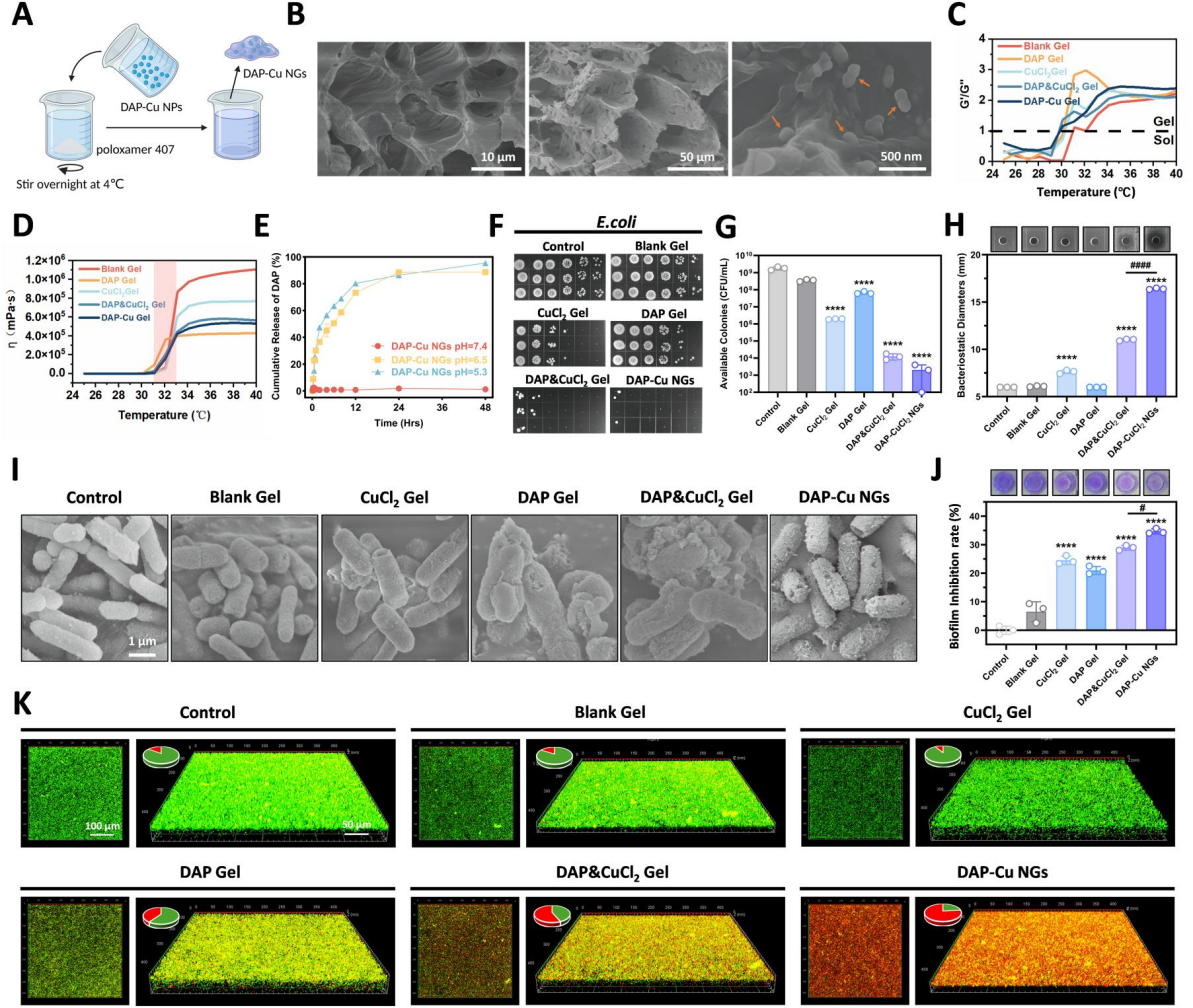
(**A**) Schematic diagram of the preparation of DAP-Cu NGs. (**B**) SEM morphology analysis of empty NGs (**left**) and DAP-Cu NGs (**middle and right**). Scale bar = 10 μm, 50 μm and 500 nm, respectively. (**C**) *G*′/*G*″ vs. temperature curves of DAP-Cu NGs. (**D**) Viscosity–temperature curve of DAP-Cu NGs. (**E**) Release curves of DAP-Cu NGs in different media. (**F**) The inhibition results of DAP-Cu NGs on *E. coli* and (**G**) quantitative analysis. (**H**) The inhibition zone results of DAP-Cu NGs against *E. coli*. (**I**) The effect of DAP-Cu NGs on the morphology of *E. coli*. (**J**) Crystal violet staining was used to evaluate the inhibition of DAP-Cu NGs on *E. coli* biofilm. (**K**) CLSM was used to evaluate the destruction of DAP-Cu NGs on *E. coli* biofilm. All data are expressed as mean ± SD (*n* = 3 per group). Statistical analysis was performed by two-tailed Student’s *t*-test (^####^*P* ≤ 0.0001) and one-way ANOVA (****P ≤ 0.0001).

SEM imaging showed that DAP-Cu NPs were distributed within the porous network structure of the poloxamer hydrogel through simple mixing (Figure 3A and B). At low magnification, the gel skeleton exhibited a typical porous morphology; at high magnification, nanoparticles were observed embedded within the pores. Rheological tests revealed the phase transition characteristics of the gel. The *G*′/*G″* ratio of each group of hydrogels showed a sudden change during the temperature increase, indicating that the sol–gel transition occurred in the 30–32°C range (Figure 3C). Compared with Blank Gel, DAP Gel and CuCl_2_ Gel, DAP-Cu NGs completed the transition from sol to gel at about 30°C, and the transition temperature was slightly lower and closer to the physiological temperature. The viscosity change curve verified this result. Viscosity measurements showed that all groups exhibited a marked increase in viscosity within the phase transition interval, with DAP-Cu NGs showing the most pronounced viscosity increase at the lowest temperature (Figure 3D). A prolonged release of DAP was observed over 48 h at both pH 6.5 and 5.3 (Figure 3E), indicating that the hydrogel matrix effectively modulates the initial burst release from nanoparticles into a prolonged release profile. This hierarchical release behavior suggests that while DAP-Cu NPs undergo rapid pH-triggered dissociation, the poloxamer network provides a secondary diffusion barrier that enables sustained drug availability in the wound microenvironment (Figure 3E). In contrast, DAP Gel (control) exhibited rapid release, with over 90% of DAP released within the first 6 h across all pH conditions (Supplementary Figure S4A).

In the antibacterial experiment, DAP-Cu NGs showed the strongest bactericidal effect. The results of colony forming unit counts showed that the number of bacteria in the control group and the Blank Gel group did not decrease significantly, and CuCl_2_ Gel and DAP Gel had certain inhibitory effects on bacteria, but the effect was limited; in contrast, the number of colonies in the DAP-Cu NGs group was almost completely cleared, showing significant bactericidal activity brought about by the synergistic effect (Figure 3F and G). This result was further supported by CLSM images with live/dead staining and corresponding quantitative analysis (Supplementary Figure S5A and B). The zone of inhibition experiment further verified this result: the inhibition zone diameter of DAP-Cu NGs was the largest, and significantly exceeded that of DAP Gel and CuCl_2_ Gel (Figure 3H). SEM imaging revealed the morphological changes of bacteria after different treatments (Figure 3I). The bacteria in the control group and the Blank Gel group maintained a complete rod-shaped morphology with a smooth surface; the bacteria in the CuCl_2_ Gel group and the DAP Gel group showed slight shrinkage, but the overall structure was still intact. The bacteria in the DAP-Cu NGs group, on the other hand, were very damaged, with ruptured and collapsed membranes. Biofilm inhibition was assessed using crystal violet staining. DAP-Cu NGs treatment resulted in the lowest residual biofilm mass, significantly lower than that of all other treatment groups (Figure 3J). CLSM imaging further verified this result from the three-dimensional structure (Figure 3K). The control group and the Blank Gel group showed a large number of green live bacteria signals, while the DAP-Cu NGs group was dominated by red dead bacteria. This indicates that DAP-Cu NGs can not only kill planktonic bacteria, but also effectively prevent bacterial adhesion and biofilm formation.

DAP-Cu NGs also exhibited excellent antibacterial effects in the Gram-positive *S. aureus* model. The results of colony counting (Supplementary Figure S4B and C) showed that the DAP-Cu NGs group almost completely inhibited the growth of bacteria, while a large number of colonies remained in the single drug group. The zone of inhibition assay (Supplementary Figure S4E and F) showed that the DAP-Cu NGs group had the largest zone of inhibition, which was much larger than that of the other groups. SEM imaging (Supplementary Figure S4D) showed that the surface structure of *S. aureus* was destroyed after DAP-Cu NGs treatment. Crystal violet staining (Supplementary Figure S4G and H) also showed consistent biofilm inhibition effects with *E. coli*.

### DAP-Cu NGs promote the accelerated healing and tissue reconstruction of infected wounds while inhibiting bacteria

**Figure 4:**
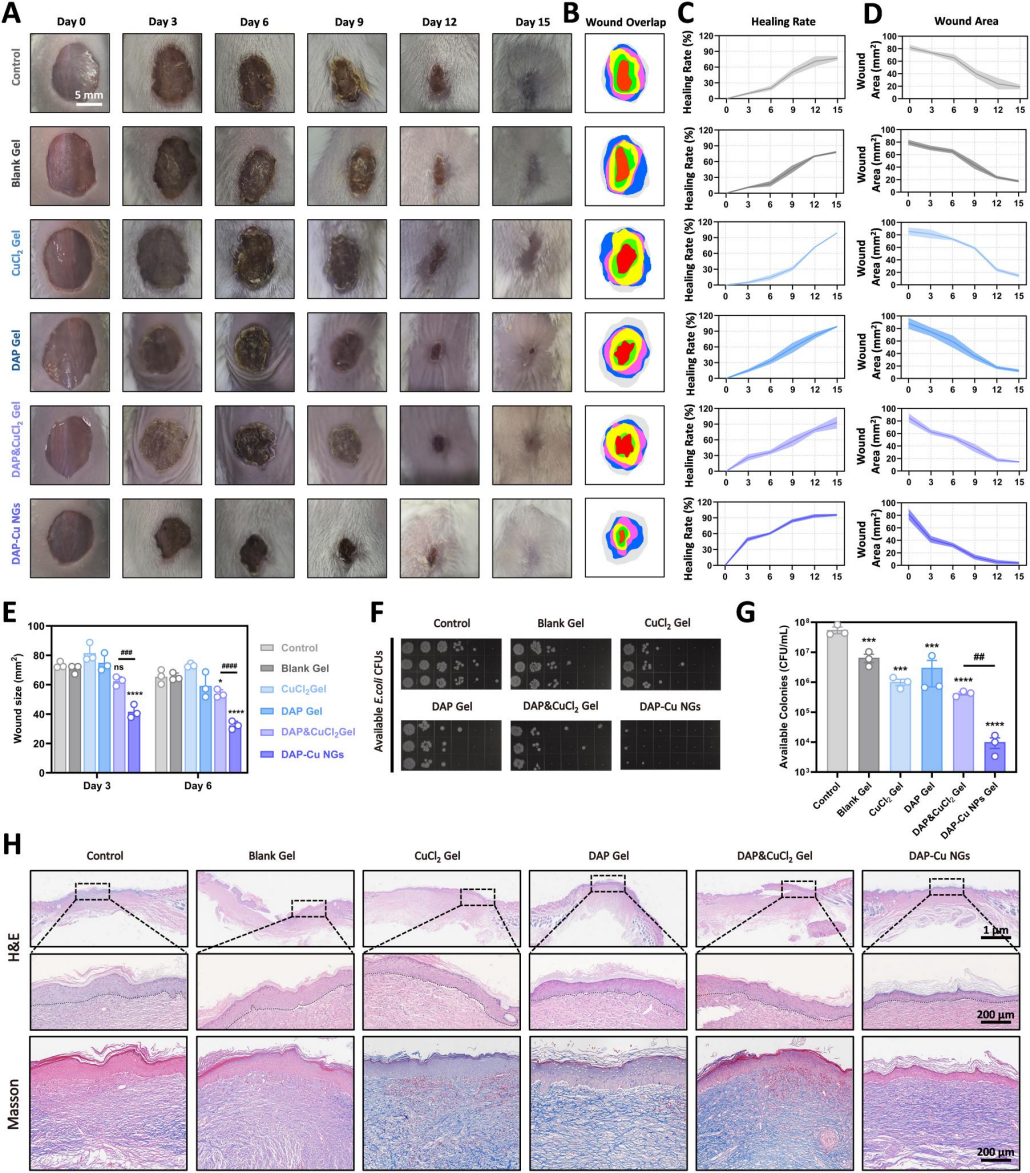
Evaluation of the effect of DAP-Cu NGs on the *E. coli*-infected wound model. (**A**) Photos of the effect of DAP-Cu NGs and its components on wound healing at different time points. (**B**) The overlapped area of wound surface at different time points after treatment with DAP-Cu NGs and its components. (**C**) The healing rate curve and (**D**) wound area curve of DAP-Cu NGs and its components after treatment of the wound during the entire treatment evaluation period. (**E**) The wound area was compared and analysed on the third and sixth days after the wound was treated with DAP-Cu NGs and its components. (**F**) Photographs and (**G**) quantitative analysis of the number of *E. coli* colonies in the wound exudate after the wound was treated with DAP-Cu NGs and its components. (**H**) After treatment, the stained sections of the wound were treated with DAP-Cu NGs and its components. All data are expressed as mean ± SD (*n* = 3 per group). Statistical analysis was performed by two-tailed Student’s *t*-test (^##^*P* ≤ 0.01, ^###^*P* ≤ 0.001 and ^####^*P* ≤ 0.0001) and one-way ANOVA (*P ≤ 0.05, ***P ≤ 0.001 and ****P ≤ 0.0001, ns means not significance).

Wound healing was evaluated over 15 days in an *E. coli*-infected full-thickness skin defect model. The continuous representative photographs showed that the wound area of the control group and the Blank Gel group shrank slowly within 15 days, showing the typical characteristics of infectious non-healing (Figure 4A). The CuCl_2_ Gel and DAP Gel groups were slightly improved compared with the control group, but there were still obvious necrotic tissues and exudates. In contrast, the DAP&CuCl_2_ Gel and DAP-Cu NGs groups showed significant healing effects, especially the DAP-Cu NGs group, which showed obvious wound contraction from day 3, and near-complete closure by day 12–15, the skin surface recovered to near integrity. The visual comparison of wound area further confirmed this trend, and the area (residual wound) of DAP-Cu NGs group was significantly smaller than that of other groups (Figure 4B). Quantitative analysis showed that the DAP-Cu NGs group had the highest wound healing rate (Figure 4C) and the fastest wound area reduction (Figure 4D), which was significantly better than other groups. The wound area on the third and sixth days was further plotted separately (Figure 4E).

In order to further evaluate the antibacterial ability of DAP-Cu NGs in the infection model, we took the wound exudate on day 3 of modeling and performed bacterial culture. Colony counting revealed abundant *E. coli* colonies in the control and Blank Gel groups (Figure 4F and G). Moderate reductions in bacterial counts were observed in the CuCl_2_ Gel and DAP Gel groups. The DAP&CuCl_2_ Gel group exhibited enhanced antibacterial effects, while the DAP-Cu NGs group showed the highest efficacy, achieving near-complete inhibition of bacterial growth. These findings are consistent with the *in vitro* antibacterial results.

To verify the regeneration of wound tissue, skin samples were taken for histological staining at the end of the experiment (Figure 4H). The results of H&E staining showed that there were extensive epidermal defects and inflammatory cell infiltration in the control group and the Blank Gel group, and the dermal structure was disordered. Although the CuCl_2_ Gel and DAP Gel groups had some new epidermis formed, the inflammatory reaction was still obvious and the healing was incomplete. The DAP&CuCl_2_ Gel group showed improved epidermal continuity and reduced inflammatory cells. The most significant was observed in the DAP-Cu NGs group, where the epidermis had basically covered the wound; the dermal structure was clear and the inflammatory cells were significantly reduced, showing the regenerative characteristics of normal skin. Masson staining further revealed the collagen deposition. The collagen fibers in the control group and the Blank Gel group were sparse and disordered; the collagen deposition in the CuCl_2_ Gel group and the DAP Gel group increased, but it was still irregular. The collagen fibers in the DAP&CuCl_2_ Gel group were relatively continuous, while the DAP-Cu NGs group showed the most ideal performance. The collagen fibers were dense and arranged in an orderly manner, suggesting that it not only accelerated wound closure, but also promoted the reconstruction of skin tissue structure.

**Figure 5:**
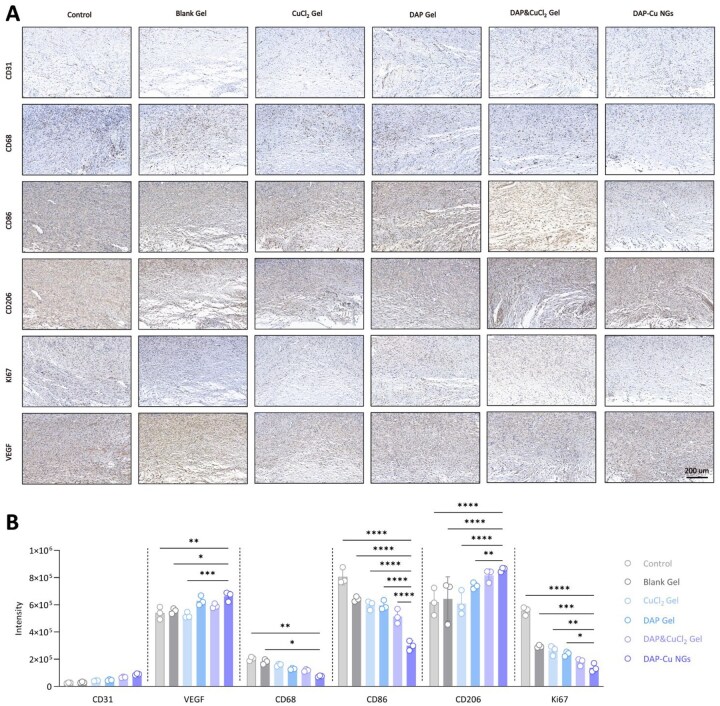
Immunohistochemical analysis of angiogenesis, macrophage polarization and cell proliferation in infected wounds on day 15. (**A**) Representative immunohistochemical staining images and (**B**) quantitative analysis showing the expression of CD31, VEGF, CD68, CD86, CD206 and Ki67 in wound tissues from different treatment groups: control, Blank Gel, CuCl_2_ Gel, DAP Gel, DAP&CuCl_2_ Gel and DAP-Cu NGs. Scale bar = 200 μm. Quantitative data are presented as mean ± SD (*n* = 3 per group). Statistical significance was determined by two-way ANOVA: *P < 0.05, **P < 0.01, ***P < 0.001 and ****P < 0.0001 vs. indicated groups.

To further elucidate the molecular mechanisms by which DAP-Cu NGs promote tissue regeneration, immunohistochemical staining was performed on wound tissues at day 15 post-treatment to assess markers associated with angiogenesis (CD31, VEGF), macrophage infiltration and polarization (CD68, CD206) and cell proliferation (Ki67) (Figure 5A and B). 4-HNE staining, a marker of lipid peroxidation, revealed elevated oxidative stress levels in the control and Blank Gel groups, which were further increased in the CuCl_2_ Gel group, indicating pronounced oxidative damage induced by free copper exposure. In contrast, oxidative stress was reduced in the DAP Gel and DAP&CuCl_2_ Gel groups, while the DAP-Cu NGs group exhibited the lowest level of 4-HNE staining, suggesting effective suppression of lipid peroxidation through coordination-mediated modulation of copper bioactivity (Supplementary Figure S6A and B). CD31 staining revealed a limited number of CD31-positive microvessels in the control and Blank Gel groups. A slight increase in CD31 expression was observed in the CuCl_2_ Gel group and the DAP Gel group. Notably, the DAP&CuCl_2_ Gel group exhibited a marked enhancement in CD31-positive signals, while the DAP-Cu NGs group showed the most pronounced CD31 expression. The trend in VEGF staining was consistent with that of CD31, with the strongest VEGF-positive expression observed in the DAP-Cu NGs group. CD68 staining demonstrated substantial infiltration of CD68-positive cells in the control and Blank Gel groups. All treatment groups showed a reduction in CD68-positive cell numbers, with the most significant decrease observed in the DAP-Cu NGs group. In contrast, CD206 staining revealed minimal expression in the control group, a slight increase in the CuCl_2_ Gel and DAP Gel groups, a significant elevation in the DAP&CuCl_2_ Gel group, and the highest level of CD206 expression in the DAP-Cu NGs group. Furthermore, the macrophage polarization state was quantitatively evaluated using the CD86/CD206 ratio, which was significantly lower in the DAP-Cu NGs group compared to other groups (Supplementary Figure S7). Ki67 staining indicated that Ki67-positive cells in the control and blank hydrogel groups were primarily localized in regions of inflammatory infiltration. An increase in Ki67 expression was observed in both the CuCl_2_ Gel group and the DAP Gel group. However, the Ki67-positive rate in the DAP-Cu NGs group was lower than that in the DAP&CuCl_2_ Gel group. The coordinated upregulation of CD31, VEGF and CD206, along with the downregulation of CD68, indicates that DAP-Cu NGs-treated wounds have transitioned into a relatively mature phase of tissue remodeling.

To evaluate the *in vivo* safety of DAP-Cu NGs, we performed systematic tests from three aspects: overall state, hematological indicators and pathological changes of major organs. During the treatment, the body weight of mice in each group showed a normal growth trend, and there was no significant difference between the DAP-Cu NGs group and the control group (Supplementary Figure S8A). The results of the hemolysis experiment **(**Supplementary Figure S8B**)** showed that the hemolysis rate of DAP-Cu NGs and other control groups was far less than 5%, indicating that the system had good safety in the blood environment. Further analysis of blood routine and biochemical indicators (Supplementary Figure S8C) showed that each parameter was within the normal range, and there was no statistically significant difference between the groups, suggesting that DAP-Cu NGs would not cause significant liver and kidney function damage or hematological abnormalities. The H&E staining results of the main organs (Supplementary Figure S8D) showed that the heart, liver, spleen, lung, kidney and other tissues were intact, without inflammatory infiltration, necrosis or fibrosis. In summary, DAP-Cu NGs have good biocompatibility and *in vivo* safety at the dose and administration cycle in this study.

## Discussion

The difficulty in healing infected wounds arises not only from persistent pathogens but also from the disruption of the regenerative microenvironment, including sustained inflammation, impaired angiogenesis and reduced cell migration. Therefore, effective therapies should go beyond bacterial eradication to restore intrinsic regenerative capacity. Based on this understanding, infection control is positioned as an ‘enabling step’ rather than a standalone endpoint, while emphasizing the intrinsic pro-regenerative bioactivity of the material. Accordingly, DAP-Cu NGs were rationally designed as a multifunctional nanocomposite hydrogel integrating environmental normalization with direct cellular modulation.

The design of DAP-Cu NGs leverages coordination chemistry to achieve two key functions: reducing Cu^2+^-associated cytotoxicity via confinement and enabling pH-responsive release. The system remains stable under physiological conditions but rapidly dissociates in acidic infection environments, allowing localized activation. Importantly, the release behavior follows a hierarchical mechanism. At the nanoparticle level, protonation-induced dissociation produces an initial burst release that supports early antibacterial action, while the hydrogel matrix provides a secondary diffusion barrier for sustained drug availability. This dual-stage release profile aligns with the dynamic needs of infected wounds, combining rapid bacterial clearance with subsequent microenvironment regulation.

From a mechanistic perspective, copper in this system functions as a coordination-regulated bioactive element rather than a freely diffusing cytotoxic agent. Compared to free Cu^2+^, which induces nonspecific oxidative damage, coordination confinement enables controlled modulation of oxidative activity. This is supported by reduced cytotoxicity *in vitro* and significantly lower 4-HNE levels *in vivo*, indicating that oxidative stress is not indiscriminately amplified but maintained within a regulated range. Differential transcriptomic responses further support this model, with bacterial cells showing metal-stress activation and host cells exhibiting antioxidant and homeostasis-related pathways. Together, these findings suggest that coordination chemistry reprograms copper bioactivity toward a more selective mode, enabling antibacterial efficacy while minimizing collateral tissue damage. However, this interpretation is based on functional evidence, and further studies on copper distribution and ROS dynamics are needed.

At the antibacterial level, DAP-Cu NGs exhibit synergistic effects. Transcriptomic analysis indicates that DAP and Cu^2+^ jointly disrupt bacterial metabolism, metal ion homeostasis and DNA repair, resulting in enhanced bactericidal activity and reduced resistance potential. In addition, the system effectively disrupts established biofilms and inhibits early biofilm formation, thereby removing physical barriers to tissue regeneration.

Importantly, the regenerative effects observed are not solely secondary to infection control but also arise from the intrinsic bioactivity of the DAP-Cu system. In bacteria-free conditions, transcriptomic analysis revealed upregulation of pathways related to cell migration, extracellular matrix remodeling and angiogenesis, indicating direct pro-regenerative activity. This is supported by enhanced cell migration *in vitro* and increased angiogenesis and M2-type macrophage polarization *in vivo*. These findings support a dual mechanism in which infection control restores a permissive environment, while the material directly promotes tissue repair.

DAP-Cu NGs also regulate the immune microenvironment by promoting macrophage polarization from the pro-inflammatory M1 phenotype to the pro-repair M2 phenotype, facilitating the transition from inflammation to tissue regeneration. At the tissue level, the material enhances keratinocyte and fibroblast migration, promotes neovascularization and improves collagen organization, ultimately leading to more effective wound healing. Notably, transcriptomic findings are supported by functional validation, including migration assays, cytokine analysis and immunohistochemistry, establishing a direct link between gene regulation and biological outcomes.

Compared with conventional wound treatments, such as silver-based dressings that primarily rely on antimicrobial activity, DAP-Cu NGs offer a distinct advantage by integrating infection control with active regulation of the regenerative microenvironment. This multifunctional strategy addresses multiple pathological features simultaneously and shows potential for treating chronic non-healing wounds.

This study has limitations. The *in vivo* model employed an acute mono-species infection, which does not fully represent the complexity of chronic wounds. However, this simplified model was intentionally used as a mechanistically controlled system to decouple antibacterial and regenerative effects, consistent with stepwise evaluation strategies in biomaterials research. Future studies will incorporate polymicrobial (e.g. *S. aureus* + *P. aeruginosa*) and impaired healing models, as well as further investigate the long-term safety and *in vivo* behavior of copper-containing systems.

## Conclusion

In summary, this study demonstrates that the impaired healing of infected wounds arises not only from bacterial burden but also from disruption of the regenerative microenvironment. By positioning infection control as an enabling step rather than a standalone endpoint, we developed a coordination chemistry-driven nanocomposite hydrogel (DAP-Cu NGs) that integrates antibacterial activity with direct modulation of host regenerative processes.

Through coordination-mediated confinement and pH-responsive activation, the system enables controlled copper bioactivity, achieving effective bacterial clearance while limiting excessive oxidative damage to host tissues. In parallel, DAP-Cu NGs promote macrophage polarization toward a pro-repair phenotype and enhance angiogenesis and tissue remodeling, supporting wound regeneration through a dual mechanism of environmental normalization and intrinsic bioactivity.

These findings highlight a design strategy that couples infection control with regenerative modulation in a single platform. While further validation in clinically relevant models is required, this work provides a proof-of-concept framework for the development of next-generation biomaterials targeting complex infected wounds.

## Supplementary Material

rbag094_Supplementary_Data

## Data Availability

The data that support the findings of this study are available from the corresponding author upon request.
