# Supplementary material for: Coordination-based nanocomposite hydrogel promotes tissue regeneration under infection-compromised conditions
Source: Regen Biomater. 2026 May 12;13:rbag094. doi: 10.1093/rb/rbag094 (PMC13228148; doi:10.1093/rb/rbag094)
Supplement: rbag094_Supplementary_Data [file rbag094_supplementary_data.docx]

**Coordination-based Nanocomposite Hydrogel Promotes Tissue Regeneration Under Infection-Compromised Conditions**

Kailing Yu ^1,*^, Jia Zhong ^1,*^, Yilin Ma ^1^, Jia Li ^1^, Yinhui Wei ^1^, Hangsheng Zheng ^1^, Fanzhu Li ^1,#^, Lai Jiang ^1,#^.

1. School of Pharmaceutical Sciences, Zhejiang Chinese Medical University, Hangzhou, China, 31140

* Co-first authorship: These authors have contributed equally to this work and share first authorship

# Correspondence: Fanzhu Li [lifanzhu@zcmu.edu.cn](mailto:lifanzhu@zcmu.edu.cn) ; Lai Jiang [laijiang.sps@zcmu.edu.cn](mailto:laijiang.sps@zcmu.edu.cn)


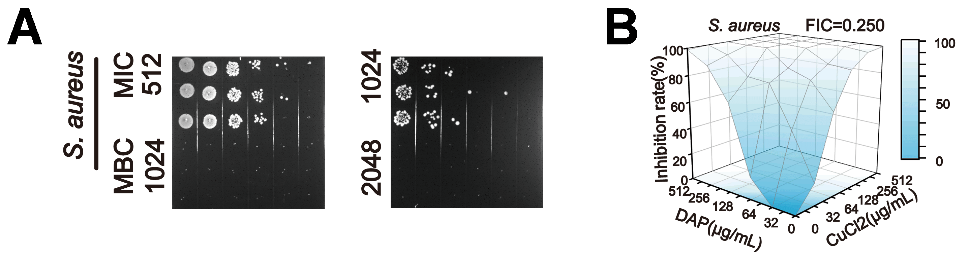


**Supplementary Figure 1. (A)** The antibacterial effects of DAP and Cu^2+^ on *S. aureus*, respectively. **(B)** Synergistic antibacterial results of DAP and Cu^2+^ on *S. aureus*.

**
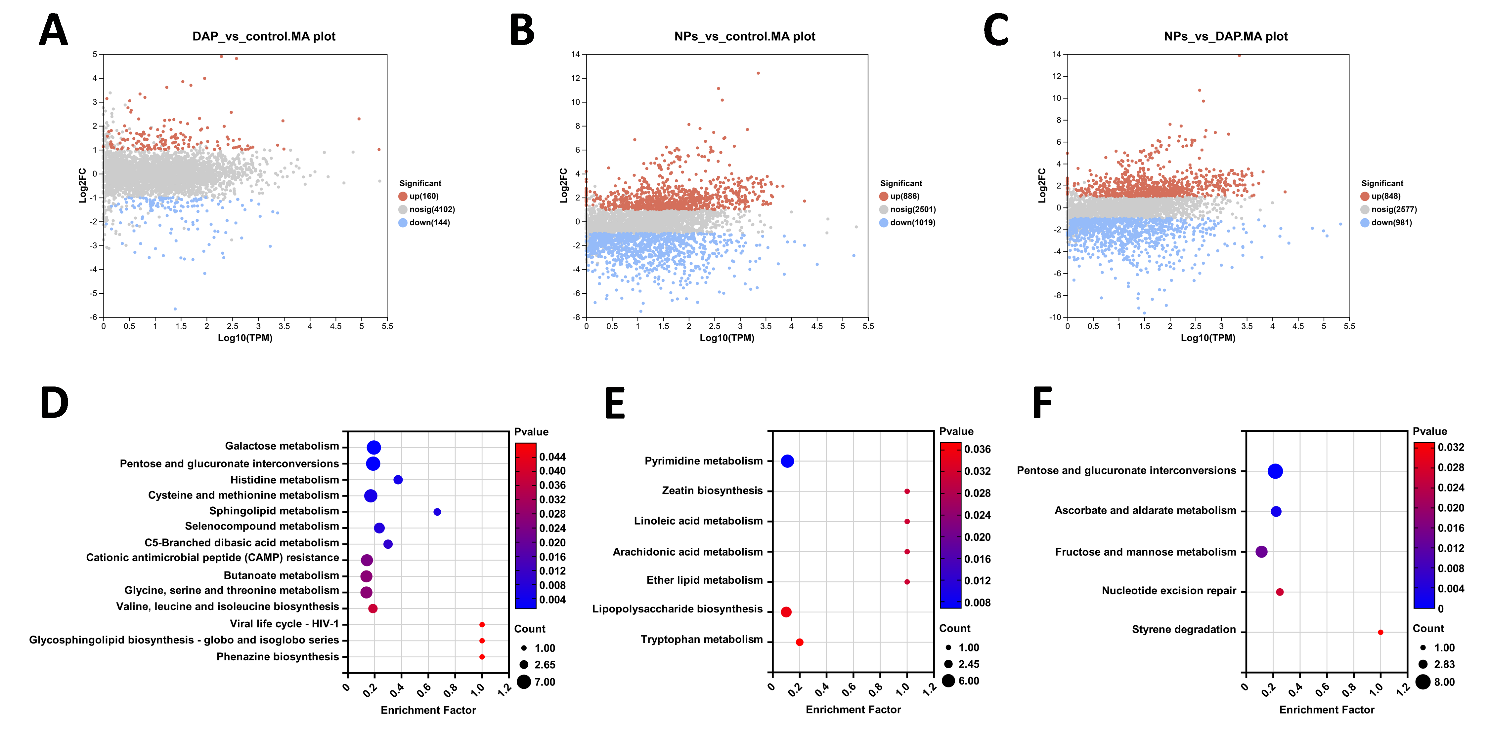
**

**Supplementary Figure 2. Transcriptomic profiling of *E. coli* treated with DAP and DAP-Cu NPs. (A-C)** Differentially expressed genes in *E. coli* after treatment with DAP or DAP-Cu NPs compared with the control group, as determined by RNA-seq analysis. **(D)** KEGG pathway enrichment analysis of the overlapping DEGs shared by the DAP and DAP-Cu NPs treatment groups. **(E)** KEGG pathway enrichment analysis of DEGs uniquely up-regulated by DAP-Cu NPs treatment. **(F)** KEGG pathway enrichment analysis of DEGs uniquely down-regulated by DAP-Cu NPs treatment.

**
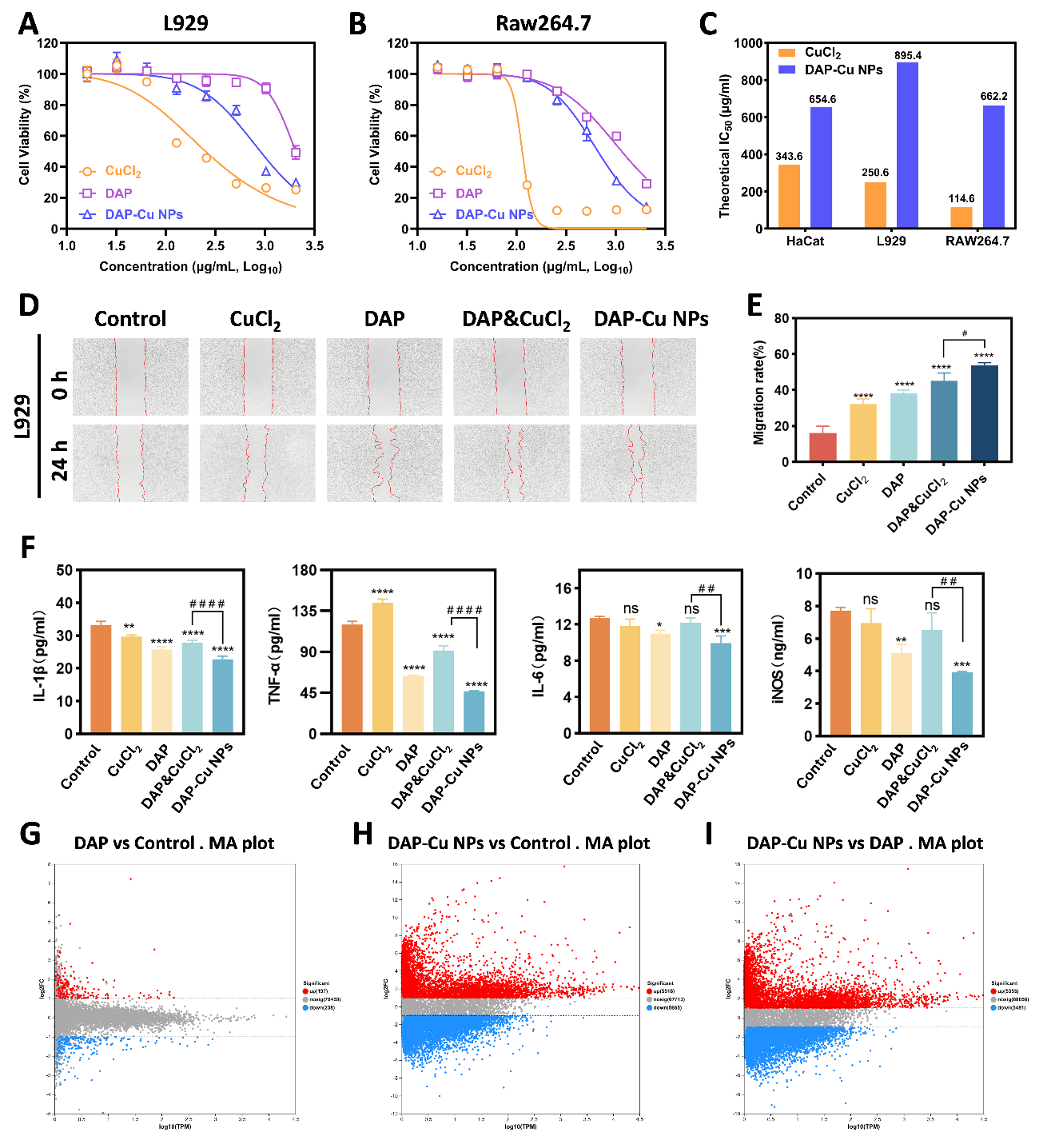
**

**Supplementary Figure 3. In vitro data on inflammatory regulation and transcriptome profiling. (A&B)** IC_50_ curves of DAP, CuCl_2_ and DAP-Cu NPs for L929 cells **(A)** and Raw264.7 cells **(B)**, respectively. **(C)** IC₅₀ values of CuCl_2_, and DAP-Cu NPs in HaCaT cells, L929 cells and RAW264.7 cells. **(D)** Photographs of the scratch wound healing assay in L929 cells treated with DAP, CuCl_2_ DAP& CuCl_2_, and DAP-Cu NPs. **(E)** Quantitative analysis of migration rate in L929 cells corresponding to panel D. **(F)** Detailed results of levels of inflammatory cytokines in the supernatant of LPS-induced RAW264.7 cells treated with DAP, CuCl₂, DAP&CuCl₂, and DAP-Cu NPs, as measured by ELISA. **(G-I) Transcriptome analysis of DEGs in HaCaT cells treated with DAP and DAP-Cu NPs. (G)** Volcano plot showing DEGs in the DAP treatment group versus control. **(H)** Volcano plot showing DEGs in the DAP-Cu NPs treatment group versus control. **(I)** Volcano plot showing DEGs between DAP and DAP-Cu NPs treatments. All data are expressed as mean ± SD. Statistical analysis was performed by Two-tailed Student’s t-test (^#^P ≤ 0.05, ^##^P ≤ 0.01, and ^####^P ≤ 0.0001) and one-way ANOVA (*p ≤ 0.05, **p ≤ 0.01, ***p ≤ 0.001, and ****p ≤ 0.0001, ns means not significance).

**Supplementary Table 1. GO enrichment analysis of overlapping DEGs in HaCaT cells co-regulated by both DAP and DAP-Cu NPs.** Red-highlighted terms represent the selected representative entries for each category as shown in Figure 2P.

| **Num of Gene** | **Term type** | **Description** | **Rich factor** | **Padjust** | **Belongs** |
| --- | --- | --- | --- | --- | --- |
| 10 | BP | angiogenesis | 2.56E-02 | 4.61E-03 | angiogenesis |
| 18 | BP | regulation of cell adhesion | 2.43E-02 | 3.08E-05 | cell adhesion |
| 11 | BP | positive regulation of cell adhesion | 2.58E-02 | 2.47E-03 |  |
| 16 | BP | cell adhesion | 1.37E-02 | 2.79E-02 |  |
| 45 | BP | cell surface receptor signaling pathway | 1.71E-02 | 2.23E-09 | cell communication / signaling pathways |
| 23 | BP | cytokine-mediated signaling pathway | 3.37E-02 | 2.96E-09 |  |
| 3 | BP | positive regulation of epidermal growth factor-activated receptor activity | 3.33E-01 | 2.04E-03 |  |
| 12 | BP | cell-cell signaling | 1.94E-02 | 8.99E-03 |  |
| 12 | BP | cell communication | 1.81E-02 | 1.54E-02 |  |
| 3 | BP | regulation of epidermal growth factor-activated receptor activity | 1.20E-01 | 2.29E-02 |  |
| 10 | BP | cell surface receptor protein tyrosine kinase signaling pathway | 1.75E-02 | 4.72E-02 |  |
| 33 | BP | cell differentiation | 1.36E-02 | 1.27E-04 | cell differentiation |
| 25 | BP | regulation of cell differentiation | 1.59E-02 | 2.30E-04 |  |
| 15 | BP | positive regulation of cell differentiation | 1.92E-02 | 2.47E-03 |  |
| 17 | BP | regulation of cell migration | 1.88E-02 | 1.16E-03 | cell migration |
| 11 | BP | positive regulation of cell migration | 2.29E-02 | 4.95E-03 |  |
| 10 | BP | regulation of epithelial cell proliferation | 3.17E-02 | 1.28E-03 | cell population proliferation |
| 15 | BP | positive regulation of cell population proliferation | 1.61E-02 | 9.95E-03 |  |
| 53 | BP | anatomical structure development | 1.37E-02 | 2.71E-08 | epidermis |
| 24 | BP | animal organ development | 1.75E-02 | 7.60E-05 |  |
| 13 | BP | tissue development | 1.93E-02 | 5.58E-03 |  |
| 13 | BP | system development | 1.68E-02 | 1.66E-02 |  |
| 16 | CC | external encapsulating structure | 2.23E-02 | 1.11E-04 | extracellular matrix & basement membrane |
| 16 | CC | extracellular matrix | 2.23E-02 | 1.11E-04 |  |
| 13 | CC | collagen-containing extracellular matrix | 2.46E-02 | 3.60E-04 |  |
| 5 | CC | basement membrane | 4.39E-02 | 1.55E-02 |  |
| 6 | MF | extracellular matrix structural constituent | 4.20E-02 | 2.97E-02 |  |
| 8 | BP | extracellular matrix organization | 2.16E-02 | 4.29E-02 |  |
| 8 | BP | extracellular structure organization | 2.14E-02 | 4.42E-02 |  |
| 3 | BP | digestive tract morphogenesis | 3.00E-01 | 2.52E-03 | morphogenesis |
| 8 | BP | tube morphogenesis | 3.05E-02 | 7.17E-03 |  |
| 14 | BP | anatomical structure formation involved in morphogenesis | 1.61E-02 | 1.52E-02 |  |
| 2 | BP | branch elongation of an epithelium | 2.50E-01 | 4.42E-02 |  |
| 4 | BP | response to transforming growth factor beta | 8.16E-02 | 1.12E-02 | response to growth factors |
| 7 | BP | response to growth factor | 2.67E-02 | 3.06E-02 |  |
| 3 | BP | response to fibroblast growth factor | 9.68E-02 | 3.55E-02 |  |
| 6 | BP | response to wounding | 3.30E-02 | 2.79E-02 | response to wounding |

**Supplementary Table 2. GO enrichment analysis of DEGs uniquely regulated by DAP-Cu NPs in HaCaT cells.** Red-highlighted terms represent the selected representative entries for each category as shown in Figure 2R.

| **Number** | **Term type** | **Description** | **Rich factor** | **Padjust** | **Belongs** |
| --- | --- | --- | --- | --- | --- |
| 18 | CC | actin cytoskeleton | 0.052023 | 0.002254 | actin cytoskeleton |
| 45 | MF | catalytic activity, acting on a nucleic acid | 0.038793 | 0.000356 | catalytic activity, acting on RNA |
| 30 | MF | catalytic activity, acting on RNA | 0.047393 | 0.000412 |  |
| 156 | MF | cation binding | 0.022295 | 0.035672 | cation binding |
| 174 | CC | cytosol | 0.024825 | 6.79E-06 | cytoplasm |
| 181 | CC | cytoplasm | 0.023697 | 5.10E-05 |  |
| 61 | CC | extracellular exosome | 0.02618 | 0.029038 | extracellular vesicle (exosome) |
| 61 | CC | extracellular vesicle | 0.025924 | 0.037602 |  |
| 61 | CC | extracellular membrane-bounded organelle | 0.025902 | 0.037602 |  |
| 61 | CC | extracellular organelle | 0.025902 | 0.037602 |  |
| 101 | CC | nucleoplasm | 0.025654 | 0.001265 | nuclear processes |
| 6 | CC | replication fork | 0.15 | 0.004444 |  |
| 202 | CC | nucleus | 0.021007 | 0.016128 |  |
| 11 | CC | kinetochore | 0.057292 | 0.026704 |  |
| 3 | CC | inner kinetochore | 0.272727 | 0.039252 |  |
| 2 | CC | RZZ complex | 0.666667 | 0.045836 |  |
| 120 | BP | nucleobase-containing compound metabolic process | 0.025316 | 0.006779 | nucleic acid metabolic process |
| 98 | BP | nucleic acid metabolic process | 0.026029 | 0.02112 |  |
| 363 | CC | membrane-bounded organelle | 0.021415 | 7.94E-07 | organelle assembly |
| 322 | CC | intracellular membrane-bounded organelle | 0.02098 | 4.82E-05 |  |
| 406 | CC | organelle | 0.019941 | 7.89E-05 |  |
| 375 | CC | intracellular organelle | 0.019829 | 0.000655 |  |
| 119 | CC | organelle membrane | 0.024315 | 0.001922 |  |
| 36 | BP | organelle assembly | 0.040314 | 0.005181 |  |
| 46 | CC | membrane-enclosed lumen | 0.029225 | 0.018082 |  |
| 46 | CC | organelle lumen | 0.029225 | 0.018082 |  |
| 46 | CC | intracellular organelle lumen | 0.029225 | 0.018082 |  |
| 11 | BP | regulation of calcium ion transmembrane transport | 0.078014 | 0.030814 | regulation of calcium ion transport |
| 8 | BP | regulation of calcium ion transmembrane transporter activity | 0.109589 | 0.031023 |  |
| 14 | BP | regulation of calcium ion transport | 0.061674 | 0.031023 |  |


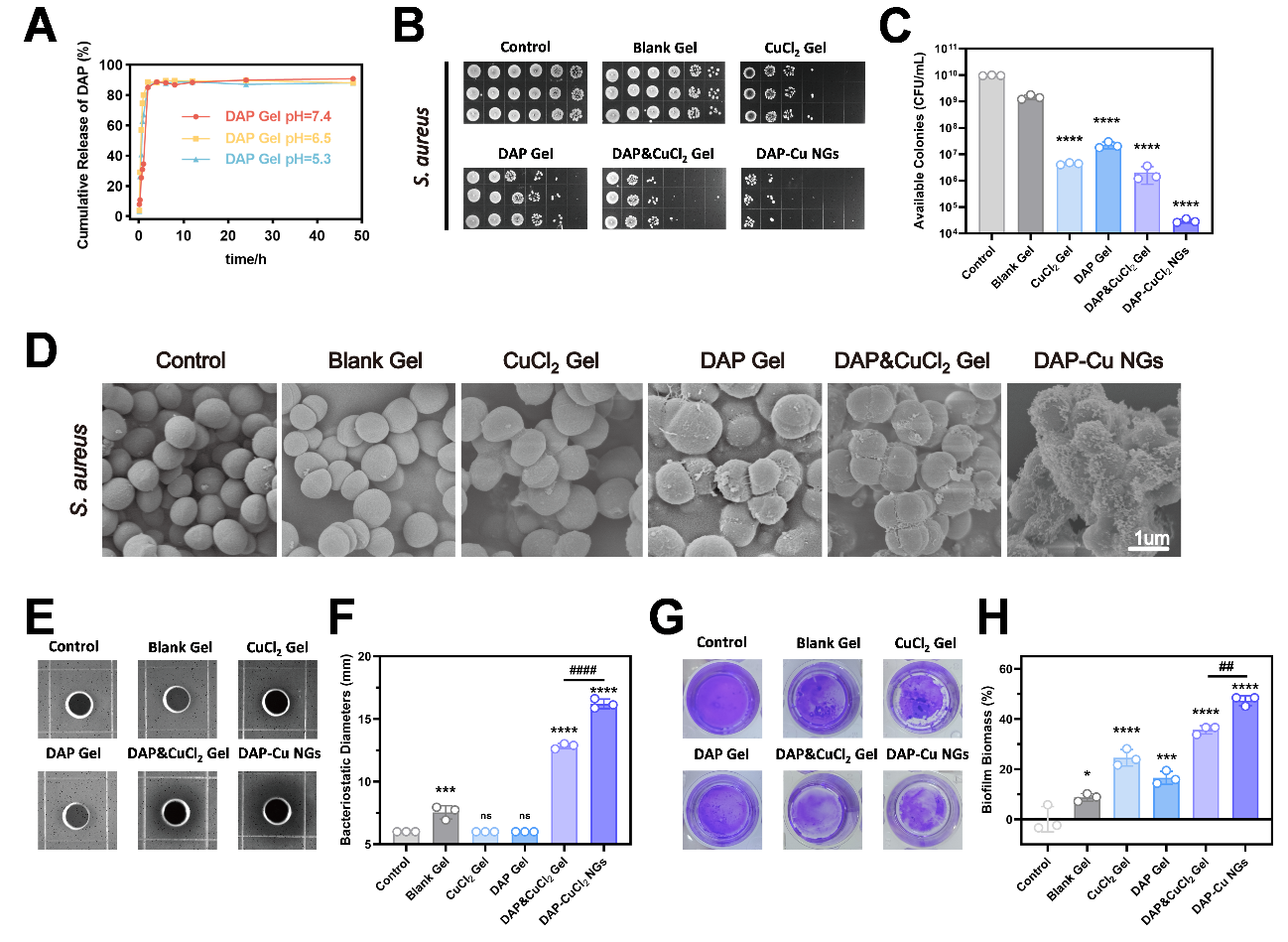


**Supplementary Figure 4. Additional characterization of DAP-Cu NGs antibacterial and antibiofilm activity against *S. aureus* and in vitro release profile of control groups. (A)** In vitro release profiles of free DAP from DAP Gel in media with different pH values (7.4, 6.5, and 5.3). **(B&C)** Photographs **(B)** and quantitative analysis **(C)** of colony-forming units showing the antibacterial activity of DAP-Cu NGs and control groups against *S. aureus.* **(D)** SEM images depicting morphological changes in *S. aureus* after treatment with DAP-Cu NGs and control groups. **(E&F)** Photographs **(E)** and quantitative analysis **(F)** of the zone of inhibition assay demonstrating the antibacterial effects of DAP-Cu NGs and control groups against *S. aureus*. **(G&H)** Crystal violet staining photographs **(G)** and quantitative analysis **(H)** evaluating the inhibition of *S. aureus* biofilm formation by DAP-Cu NGs and control groups. All data are expressed as mean ± SD. Statistical analysis was performed by Two-tailed Student’s t-test (^##^ p ≤ 0.01, ^####^ p ≤ 0.0001) and one-way ANOVA (* p ≤ 0.05, *** p ≤ 0.001, and **** p ≤ 0.0001, ns means not significance).

**
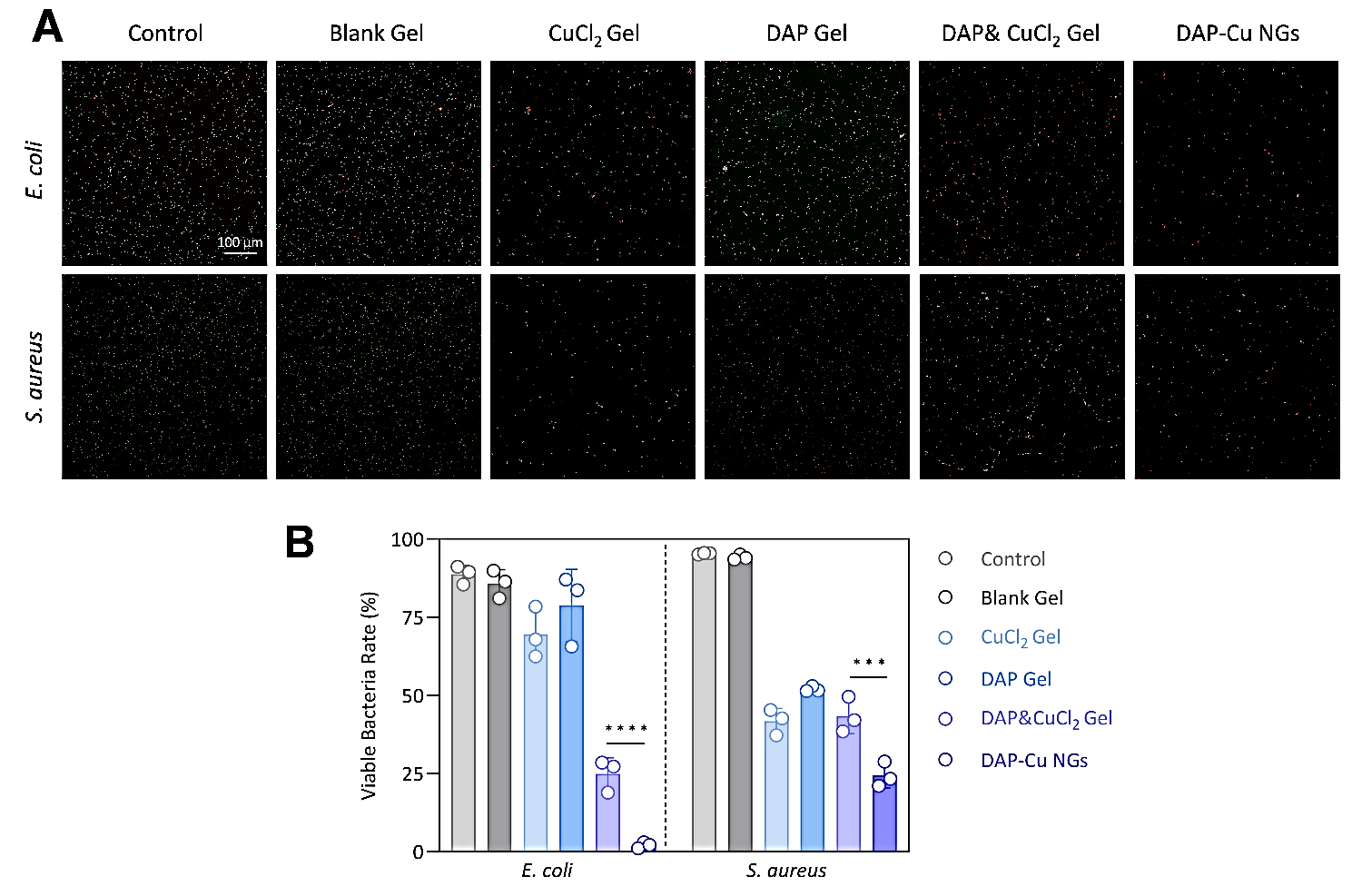
**

**Supplementary Figure 5.** **Live/dead fluorescence assay of bacteria treated with various formulations.** **(A)** CLSM images of *E. coli* and *S. aureus* stained with Live/Dead BacLight dye after exposure to the indicated treatments. Green fluorescence indicates live bacteria; red fluorescence indicates dead bacteria. Scale bar = 100 μm. **(B)** Quantitative analysis of the percentage of viable bacteria based on fluorescence intensity. Quantitative data are presented as mean ± SD (n = 3 per group). Statistical significance was determined by two-way ANOVA: ** p < 0.01.


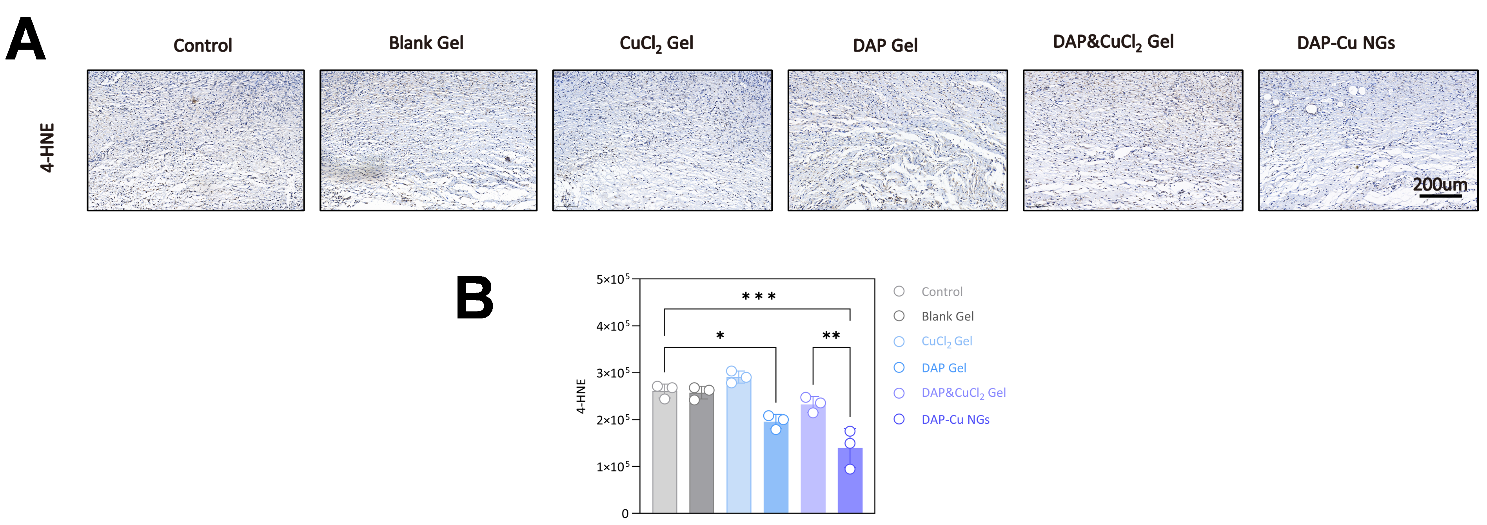


**Supplementary Figure 6. Coordination-mediated modulation of lipid peroxidation in infected wounds. (A)** Representative IHC staining images and **(B)** Quantitative analysis of 4-hydroxynonenal (4-HNE) in wound sections from different treatment groups (Control, Blank Gel, CuCl_2_ Gel, DAP Gel, DAP& CuCl_2_ Gel, and DAP-Cu NGs). Scale bar: 200 μm. Data are presented as mean ± SD (n = 3). Statistical analysis was performed using one-way ANOVA with Tukey’s multiple comparisons test. * p < 0.05, ** p < 0.01, *** p < 0.001.


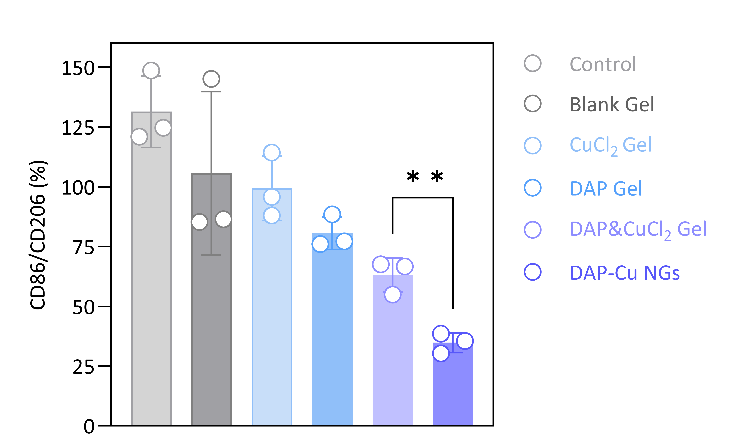


**Supplementary Figure 7. Quantitative analysis of the M1/M2 macrophage ratio in infected wounds on day 15.** The CD86/CD206 ratio was calculated based on immunohistochemical staining to evaluate macrophage polarization. Statistical significance was determined by *t*-test: ** p < 0.01 versus indicated groups.


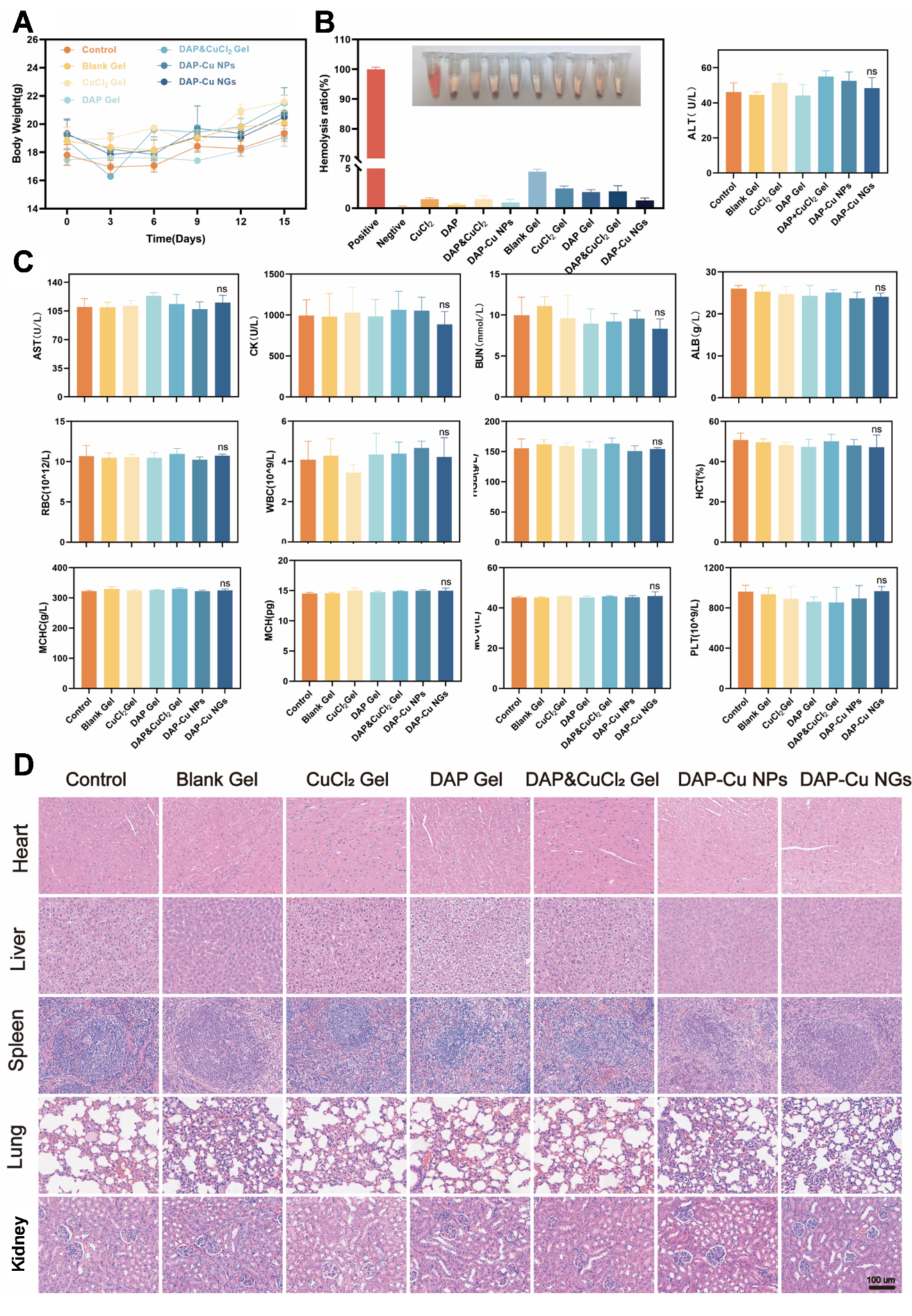


**Supplementary Figure 8. In vivo biosafety evaluation of DAP-Cu NGs. (A)** Body weight changes of Balb/c mice in each treatment group over the 15-day treatment period. **(B)** Hemolysis rates of red blood cells incubated with CuCl_2_, DAP, DAP& CuCl_2_, DAP-Cu NPs, Blank Gel, CuCl_2_ Gel, DAP Gel, DAP& CuCl_2_ Gel, and DAP-Cu NGs (negative control: saline; positive control: pure water). **(C)** Blood routine and biochemical parameters of mice in each group at the end of the treatment cycle. **(D)** Representative H&E stained sections of major organs (heart, liver, spleen, lung, and kidney) from mice in each treatment group after the 15-day treatment. All data are expressed as mean ± S.D. Statistical analysis was performed by one-way ANOVA (ns means not significance).
